# High-dimensional assessment of B-cell responses to quadrivalent meningococcal conjugate and plain polysaccharide vaccine

**DOI:** 10.1186/s13073-017-0400-x

**Published:** 2017-01-30

**Authors:** Daniel O’Connor, Elizabeth A. Clutterbuck, Amber J. Thompson, Matthew D. Snape, Maheshi N. Ramasamy, Dominic F. Kelly, Andrew J. Pollard

**Affiliations:** 1 Department of Paediatrics, Oxford Vaccine Group, University of Oxford, Churchill Hospital, Oxford, UK; 2 NIHR Oxford Biomedical Research Centre, Oxford, UK

## Abstract

**Background:**

*Neisseria meningitidis* is a globally important cause of meningitis and septicaemia. Twelve capsular groups of meningococci are known, and quadrivalent vaccines against four of these (A, C, W and Y) are available as plain-polysaccharide and protein-polysaccharide conjugate vaccines. Here we apply contemporary methods to describe B-cell responses to meningococcal polysaccharide and conjugate vaccines.

**Methods:**

Twenty adults were randomly assigned to receive either a meningococcal plain-polysaccharide or conjugate vaccine; one month later all received the conjugate vaccine. Blood samples were taken pre-vaccination and 7, 21 and 28 days after vaccination; B-cell responses were assessed by ELISpot, serum bactericidal assay, flow cytometry and gene expression microarray.

**Results:**

Seven days after an initial dose of either vaccine, a gene expression signature characteristic of plasmablasts was detectable. The frequency of newly generated plasma cells (CXCR3^+^HLA-DR^+^) and the expression of transcripts derived from *IGKC* and *IGHG2* correlated with immunogenicity. Notably, using an independent dataset, the expression of glucosamine (N-acetyl)-6-sulfatase was found to reproducibly correlate with the magnitude of immune response. Transcriptomic and flow cytometric data revealed depletion of switched memory B cells following plain-polysaccharide vaccine.

**Conclusions:**

These data describe distinct gene signatures associated with the production of high-avidity antibody and a plain-polysaccharide-specific signature, possibly linked to polysaccharide-induced hyporesponsiveness.

**Electronic supplementary material:**

The online version of this article (doi:10.1186/s13073-017-0400-x) contains supplementary material, which is available to authorized users.

## Background

Polysaccharide-encapsulated organisms are the leading causes of bacterial meningitis and pneumonia in children. *Neisseria meningitidis* is estimated to cause 500,000 serious illnesses worldwide each year [1]. Clinical outcomes of invasive infection vary by setting and strain, but even in resource rich countries they are poor, with permanent neurological sequelae common and up to 10% of those affected dying [1]. While susceptibility to invasive meningococcal disease is not completely understood, an inverse relationship is seen with the prevalence of complement-dependent serum bactericidal assay (SBA) titres [2], and the levels of bactericidal antibody correlate with post-immunisation population protection [3].

Meningococci comprise 12 capsular groups, six of which, A, B, C, W, X and Y, are responsible for the vast majority of meningococcal disease [4]. Immunisation with meningococcal capsular polysaccharides (with the exception of capsular group B polysaccharide) induces capsular group-specific SBA activity [5]. However, meningococcal polysaccharide vaccines provide only short-term protection in adults, have limited immunogenicity in early childhood and have been associated with hyporesponsiveness following subsequent doses [6, 7]. These shortcomings have been attributed to the T cell-independent nature of responses to polysaccharides that do not drive the formation of immunological memory. Chemical conjugation of the polysaccharide to a protein carrier directs T-dependent responses [8]. Quadrivalent meningococcal vaccines (MenACWY) are licensed in two forms: plain polysaccharide or polysaccharide conjugated to a carrier protein (CRM_197_ non-toxic mutant of diphtheria toxin or tetanus toxoid). While both of these vaccines are immunogenic in adults, the B-cell responses to these vaccines have not been described in detail. In particular, the mechanisms underlying hyporesponsiveness (inferior responses to subsequent doses), which is evident after plain-polysaccharide but not protein-conjugated vaccines, have yet to be elucidated [9].

We have previously shown pneumococcal polysaccharide and conjugate vaccines produce distinct B-cell responses in adults, with the former depleting memory and B1b-cell subsets [6]. Here, we apply contemporary systems biology tools (gene expression, multi-parameter flow cytometry and cellular and serological assays) to describe B-cell responses to the quadrivalent plain-polysaccharide vaccine (MenACWY-PS) and quadrivalent conjugate vaccine (MenACWY-CRM).

## Methods

### Study participants and vaccines

Healthy adult volunteers (30–70 years of age) were randomly assigned into four groups to be immunised with intramuscular MenACWY-CRM (group 1; *n* = 5), intramuscular MenACWY-PS (group 2; *n* = 5), subcutaneous MenACWY-PS (group 3; *n* = 5), or one-fifth dose intramuscular MenACWY-PS (group 4; *n* = 5). Allocation to groups was performed on a 1:1:1:1 basis, generated by computer randomisation with block size of 4 and concealed in sequentially labelled opaque envelopes. This study was open labelled with randomisation occurring at the point that the opaque envelope corresponding to the designated study number was opened (just prior to first vaccination). MenACWY-CRM (Menveo®; Novartis Vaccines, Bellario-Rosia, Italy) consisted of *N. meningitidis* capsular groups A, C, W and Y oligosaccharides (10, 5, 5 and 5 μg, respectively) individually conjugated to CRM_197_ carrier protein. MenACWY-PS (ACWYVax®; GlaxoSmithKline, Rixensart, Belgium) consisted of *N. meningitidis* capsular groups A, C, W and Y capsular polysaccharides (50 μg each serogroup).

All participants then received intramuscular MenACWY-CRM vaccine 28 days after the initial meningococcal vaccine. Blood samples were taken at study days 0 (prior to the first vaccine), 7, 28 (prior to the second vaccine), 35 and 56. One participant withdrew from the study. The study was open labelled, with both clinical staff members and participants aware of the vaccine received; however, laboratory staff members were blinded to the group allocations. An Oxfordshire research ethics committee approved this study (NRES Committee South Central Oxford C 12/SC/0275).

### Transcriptomic analysis

Total RNA was extracted from 2.5 ml peripheral blood collected into a PAXgene™ RNA stabilisation tube, using the Blood RNA Kit (PreAnalytiX, Switzerland). These RNAs were then globin depleted using GLOBINclear™ (Thermo Fisher scientific, Massachusetts). RNA samples were randomly allocated to an Illumina® Human HT12v4.0 Expression BeadChip microarray. RNA was converted into biotin-labelled cRNA and hybridised to a BeadChip microarray. Hybridised microarrays were scanned using an Illumina® iScan scanner by the Wellcome Trust Centre for Human Genetics core facility (Oxford, UK). Sample transcript profile raw data were extracted from Illumina® GenomeStudio version 1.9.0. Negative background intensities for each array were subtracted from their respective transcript intensities prior to normalisation. Transcript intensities were normalised using robust spline normalisation [10]. Microarray data were filtered to return transcripts that were significantly different from their local background (detection *p* value of less than 0.05) in at least 60% of all the samples assessed. Variance-based statistical methods (sample transcript intensity distribution boxplots, principal component analysis and hierarchical clustering) were used to quality control microarray and check for sample outliers.

A linear model was fitted with normalised expression of each transcript as the response variable and each paired sample and vaccination status as predictors. The empirical Bayes method was then used to generate moderated t-statistics, moderated F-statistics and log-odds of differential expression using the eBayes function in the Limma R package [11].

### Gene set and blood transcriptional module analysis

Gene-set enrichment analysis (GSEA) was undertaken on the entire list of filtered transcripts, ranked by their t-statistic from Limma, using the GseaPreranked tool in the Java-based desktop application of GSEA v2.0.14 [12]. Analysis was completed using the 1910 gene sets in the ‘c7:Immunological signature’ database identified from microarray experiments of gene expression in immunological studies (http://www.broadinstitute.org/gsea/msigdb/index.jsp). Blood transcriptional module analysis was undertaken using the tmod R package on genes ranked by t-statistic; statistical testing for module expression was evaluated using the tmodCERNOtest function, which is a nonparametric test working on gene ranks [13]. Module activity scores were determined by taking the mean of the absolute log2 fold changes in gene expression.

### Gene Expression Omnibus validation cohort

We utilised a publicly available dataset, acquired from the genomics data repository Gene Expression Omnibus (GEO; http://www.ncbi.nlm.nih.gov/geo/), as a validation cohort. This dataset comprised adults vaccinated with either MenACWY-PS (*n* = 13) or MenACWY-CRM (*n* = 17), as described by Li et al. [14]. Series and platform data were downloaded from GEO using the GEOquery R package [15]. These data were robust multi-array average (RMA) normalised and analysed as previously detailed.

### Isolation of peripheral blood mononuclear cells and purification of CD19^+^ B cells

Peripheral blood mononuclear cells (PBMCs) were isolated from 35 ml heparinised whole blood using lymphoprep density centrifugation. Purified CD19^+^ B cells where then obtained by anti-CD19-magnetic bead separation (Miltenyi Biotech, UK) as per the manufacturer’s instructions using AutoMACs® (Miltenyi Biotech, UK).

### Antibodies for flow cytometric characterisation of B cells and plasma cells

Antibodies for flow cytometric characterisation of B cells and plasma cells were CD19-FITC (clone HIB19, ebioscience, UK), CD5-FITC (clone L17F12, ebioscience, UK), CD38-PE (clone HB7, ebioscience, UK), CD43-PE (clone 84-3C1, ebioscience, UK), HLA-DR-PerCPCy5.5 (clone L243, Biolegend, Cambridge Bioscience, UK), IgM-PerCPCy5.5 (clone MHM-88, Biolegend, Cambridge Bioscience, UK), CD27-PECy7 (clone 0323, ebioscience, UK), CD3-V500 (clone UCHT1, BD Biosciences, UK), CD14-V500 (clone M5E2, BD Biosciences, UK), CD16-V500 (clone 3G8, BD Biosciences, UK), CXCR3-APC (clone G02SH7, Biolegend, Cambridge Bioscience, UK), IgD-APC (clone IgD26, Miltenyi Biotech, UK) and CD20-APCH7 (clone L27, BD Biosciences, UK). Propidium iodide (PI; ebioscience, UK) was used as viability stain.

### Flow cytometric characterisation of B cells and plasma cells

Purified, CD19^+^ B cells, re-suspended in phosphate-buffered saline (PBS)-EDTA + 0.5% bovine serum albumin (BSA), were added at 2 × 10^5^ cells per well of a V-bottom, 96-well culture plate in a 50-μl volume. The B cells were then labelled with a combination of the above antibodies to give: (1) plasma cells, which were viable (PI^−^) CD3^−^CD14^−^CD16^−^ CD19^+^CD20^lo/-^CD38^hi^CD27^hi^ with subsets based on expression of CXCR3 and HLA-DR; (2) memory B cells, which were viable (PI^−^) CD3^−^CD14^−^CD16^−^ CD19^+^CD20^+^CD43^−^ CD5^−^CD27^+^ with subsets based on IgM and IgD expression (IgM^+^ only, IgM^+^IgD^+^, IgD^+^ only and switched IgM^−^IgD^−^).

The antibodies were incubated on ice in the dark for 30 minutes and then washed twice in 200 μl of PBS-EDTA + 0.5% BSA, at 250 × g for 10 minutes. The B cells were then fixed for 10 minutes with BD Cell Fix® (BD Biosciences UK) at room temperature in the dark and then washed once as above and resuspended in 200 μl PBS-EDTA-0.5% BSA. The cells were transferred to microtubes in a total volume of 350 μl PBS-EDTA-0.5% BSA and stored at 4 °C overnight. The cells were analysed on a Beckman-Coulter Cyan Flow Cytometer with 9-colour parameters. The data were acquired using Summit^TM^ software and analysed using Flow Jo® version 10.0.6 software (Tree Star, USA).

### Serum bactericidal assay

Human complement serum bactericidal assays (SBAs) for detection of meningococcal serogroups A and C were performed at Vaccine Evaluation Unit, Public Health England, Manchester. Serum samples collected at days 0, 28 and 56 were assessed for human SBA activity against serogroup A (F8238) and serogroup C (C11). In brief, twofold dilutions of heat-inactivated sera were incubated with suspensions of the aforementioned *N. meningitidis* strains and freshly thawed exogenous human complement. SBA titres were expressed as the reciprocal end point serum dilutions yielding ≥50% killing of bacterial colonies after 60 minutes of incubation compared with growth at time 0. The lower limit of quantification for the SBA assays was 4; samples without detectable SBA activity were assigned an arbitrary value of 2.

### B-cell enzyme-linked immunospot assay

PVDF 96 well plates (Millipore) were coated with 100 μl of either 5 μg/ml (capsular group A and C) of purified meningococcal polysaccharide (National Institute for Biological Standards and Control (NIBSC) 98/722 and 07/318) conjugated to 5 μg/ml methylated human albumin (NIBSC), 10 μg/mL diphtheria toxoid (Statens Serum Institut 2675) or phosphate-buffered saline (background control). Prior to cells being seeded onto the plates, all wells were blocked with complete medium.

Memory B cells were assessed using cultured ELISpot performed on blood samples collected at days 0, 28 and 56. PBMCs were suspended in R10 at a concentration of 2 × 10^6^ cells/ml. These cells were cultured with an additional 100 μl of RPMI with 10% newborn bovine serum (NBBS), *Staphylococcus aureus* Cowan strain (SAC) at 1:2500 dilution of the Pansorbin cell suspension (Calbiochem-Novabiochem), 166 ng/ml pokeweed mitogen (Sigma-Aldrich) and 3.4 μg/ml CpG oligonucleotide (InvivoGEN). The cells were incubated for 6 days at 37 °C in 5% carbon dioxide and 95% humidity, after which time they were washed and processed as described by Lazarus et al. [9].

### Quantitative real-time PCR

Total RNA, extracted from PAXgene™ tubes, was reverse transcribed to cDNA using SuperScript III Reverse Transcriptase (Thermo Fisher scientific, Massachusetts). A primer sequence specific to the spliced *XBP1* isoform was obtained from the literature, sense 5′-GGTCTGCTGAGTCCGCAGCAGG-3′ and anti-sense 5′-GGGCTTGGTATATATGTGG-3′ [16]. The last two nucleotides of the spliced isoform sense primer were modified to 5′-GGTCTGCTGAGTCCGCAGCACT-3′ to create a primer complementary to the unspliced *XBP1* isoform; specificities of these primers were demonstrated by comparison of the amplicons run on an agarose (1%) gel (Additional file 1: Figure S12). GAPDH mRNA expression was used as an internal control, sense 5′- GAAGGTGAAGGTCGGAGTC-3′ and anti-sense 5′-GAAGATGGTGATGGGATTTC-3′. The real-time PCR (RT-PCR) used the Platinum® SYBR® Green SuperMix-UDG detection system (with ROX) on a StepOnePlus™ instrument (Thermo Fisher scientific, Massachusetts). The RT-PCR settings were 95 °C for 5 minutes, followed by 40 cycles of 95 °C for 30 s, 50 °C for 30 s, 72 °C for 30 s. Samples were assayed in triplicate and the median ΔCt (cycle threshold compared with the internal control GAPDH) used in subsequent analyses.

## Results

The study design and demographics are shown in Table 1 and Additional file 2: Table S1, respectively.

**Table 1:** Overview of study time points, interventions and analyses

	Study day 0	Study day 7	Study day 21	Study day 28	Study day 35	Study day 49	Study day 56
Group							
Group 1 (n = 5)	MenACWY-CRM (i/m)			MenACWY-CRM (i/m)			
Group 2 (n = 5)	MenACWY-PS (i/m)			MenACWY-CRM (i/m)			
Group 3 (n = 5)	MenACWY-PS (s/c)			MenACWY-CRM (i/m)			
Group 4 (n = 5)	MenACWY-PS (^1^/_5_ i/m)			MenACWY-CRM (i/m)			
Immunological phenotypes							
SBA (MenA and MenC)	✔			✔			✔
B mem ELISpots (MenA, MenC, DT)	✔			✔			✔
Systems tools							
Flow cytometry	✔	✔	✔	✔	✔	✔	✔
Transcriptomics	✔	✔			✔		

*i/m* intramuscular, *s/c* subcutaneous, ^*1*^ */*_*5*_ a fifth of the standard dose was administered, *SBA* serum bactericidal assay, *B mem ELISpots* B-cell memory enzyme-linked immunospot, *DT* diphtheria toxoid

### The rise in bactericidal antibody following an initial dose of either plain polysaccharide or conjugate MenACWY vaccine is similar and is not boosted by a subsequent dose of conjugate vaccine

We evaluated B-cell responses in 20 healthy adults vaccinated with either MenACWY-CRM (group 1, intramuscular, *n* = 5) or MenACWY-PS (group 2, intramuscular, *n* = 5; group 3, subcutaneous, *n* = 5; group 4, one-fifth dose intramuscular, *n* = 5), followed by an additional dose of MenACWY-CRM 28 days later. We measured anti-meningococcal capsular group A (MenA) and C (MenC) specific SBA titers, ex vivo and cultured antibody-secreting cell (ASC) frequencies using enzyme-linked immunospot (ELISpot), following each vaccination.

**Fig. 1:**
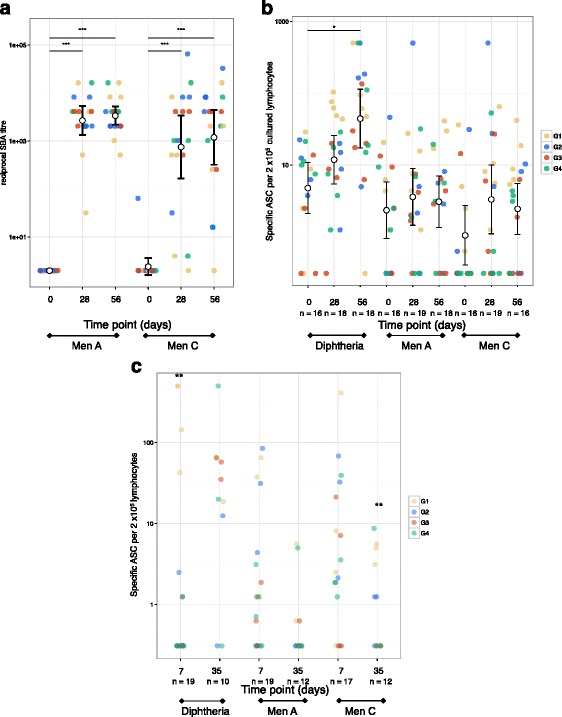
**a** MenA- and MenC-specific serum bactericidal assay titres at each study time point, with geometric mean titre (*dot*) and 95% confidence intervals (*bars*) across all groups indicated and group allocation denoted by *colour*. **b** MenA-, MenC- and diphtheria toxoid-specific cultured antibody-secreting cell (*ASC*) ELISpots at each study time point, with geometric mean concentrations (*dot*) and 95% confidence intervals (*bars*) across all groups indicated and group allocation denoted by *colour*. Group 1 (*G1*), MenACWY-CRM (day 0) + MenACWY-CRM (day 28); group 2 (*G2*), intramuscular MenACWY-PS (day 0) + MenACWY-CRM (day 28); group 3 (*G3*), subcutaneous MenACWY-PS (day 0) + MenACWY-CRM (day 28); group 4 (*G4*), one-fifth dose intramuscular MenACWY-PS (day 0) + MenACWY-CRM (day 28). **c** MenA-, MenC- and diphtheria toxoid-specific ex vivo antibody-secreting cell (*ASC*) ELISpots, 7 days after each dose of vaccine (day 7 and day 35). ***p <0.001, **p < 0.01, *p < 0.05 by Welch’s *t*-test, comparing protein-conjugated vaccine (G1) with plain-polysaccharide vaccine (G2, G3 and G4) recipients, or paired *t*-test between time points (denoted by *horizontal lines*)

The first dose of either MenACWY-CRM or MenACWY-PS elicited a rise in SBA geometric mean titres (GMTs) against MenA and MenC 28 days post-vaccination for all groups (Fig. 1a). One participant failed to produce a detectable rise in SBA titre to MenC. The second dose did not induce a further rise in SBA titres as GMTs 28 days after the first and second dose were comparable. One participant did not have a detectable SBA titre against MenC following the second dose of vaccine; this participant had a titre of 1:4 after the first dose. No statistically significant differences were seen in MenA or MenC SBA GMTs between the four vaccine groups after the first or second dose of vaccine (Fig. 1a). No statistically significant rises in MenA- or MenC-specific cultured ASCs were detected, either when vaccine groups were analysed individually or combined; however, a rise in diphtheria-specific ASCs was observed after the first dose of MenACWY-CRM (Fig. 1b). MenA- and MenC-specific ex vivo ASCs were detectable 7 days after each vaccine, with inferior MenC-specific ex vivo ASCs seen after the second dose of vaccine in those who previously received MenACWY-PS (Fig. 1c).

### Expansion of recently generated plasma cells can be detected 7 days after a first dose of MenACWY vaccine but does not distinguish polysaccharide and conjugate vaccines

**Fig. 2:**
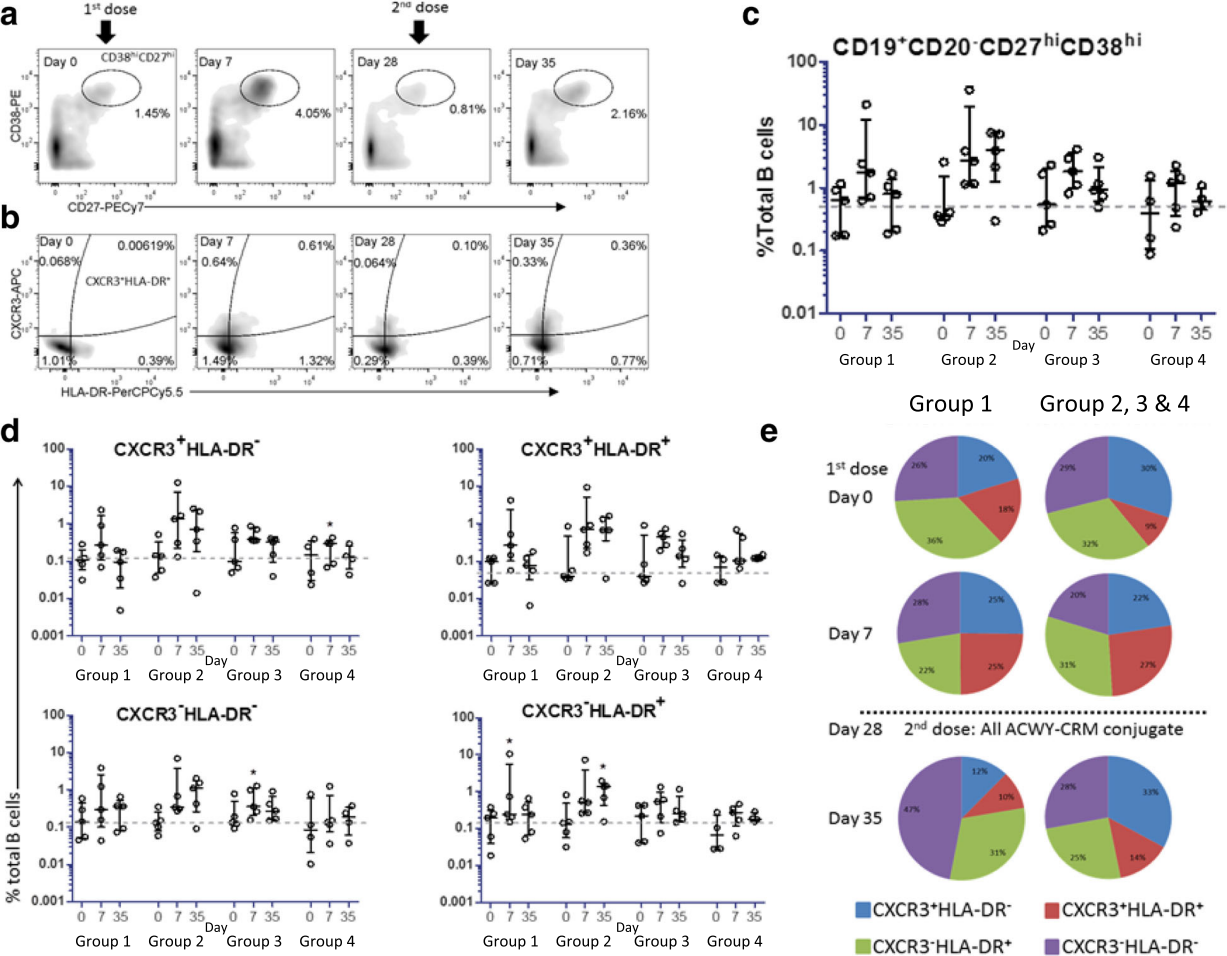
Phenotypic characterisation of peripheral blood plasma cells (PBMCs) following immunisation. Purified, CD19^+^ B cells were labelled for identification of plasma cells at baseline (day 0) and 7 days post first (day 7) and second dose (day 35). Representative plots (group 1 participant) of CD38^hi^CD27^hi^ plasma cells (**a**) and CXCR3 HLA-DR subsets (**b**) are shown for days 0, 7 and 35, with day 28 included to demonstrate a return to baseline prior to the second dose. The overall frequencies of total CD38^hi^CD27^hi^ plasma cells per group (**c**) and CXCR3 HLA-DR subsets (**d**) at baseline and 1 week after each vaccine dose are shown per group. Data are expressed as percentage of total B cells, with *bars* showing median frequency (interquartile range). ANOVA were performed within each group and the significant differences are denoted (*p = <0.05). Group 1, MenACWY-CRM/MenACWY-CRM; group 2, MenACWY-PS (intramuscular)/MenACWY-CRM; group 3, MenACWY-PS (subcutaneous)/MenACWY-CRM; group, MenACWY-PS (one-fifth dose intramuscular)/MenACWY-CRM. **e** Proportion of plasma cell subsets (percentage of total) at each study time point for the conjugate group 1 (*n* = 5) and polysaccharide groups 2, 3 and 4 (combined, *n* = 14)

Figure 2 shows the plasma cell response following immunisation across the four study groups. The total plasma cell population (CD19^+^CD20^lo^CD38^hi^CD27^hi^) rose from baseline to 7 days post-immunisation in all groups, returning to baseline by day 28 (Fig. 2a, c). CXCR3 and HLA-DR expression was used to define subsets of plasma cells; CXCR3 is a marker of chemotactic homing to bone marrow or inflamed tissue and HLA-DR is a marker of recently generated plasma cells [17]. An expansion of the CXCR3^+^HLA-DR^+^ subset 7 days post-immunisation with either MenACWY-CRM or MenACWY-PS was observed (Fig. 2e). The CXCR3^+^HLA-DR^+^ subset expanded again in MenACWY-PS + MenACWY-CRM participants (groups 2, 3 and 4) 7 days (day 35) after administration of MenACWY-CRM but not in those immunised with two doses of MenACWY-CRM (group 1). While the overall frequency of plasma cells appeared lower at day 35 in group 1 (two doses of MenACWY-CRM), the CXCR3^−^HLA-DR^−^ population appeared expanded compared with the other groups (Fig. 2d, e).

### A plasma cell gene signature is evident in whole blood 7 days after the initial dose of MenACWY vaccine with similarities and differences between plain polysaccharide and conjugate vaccines

**Fig. 3:**
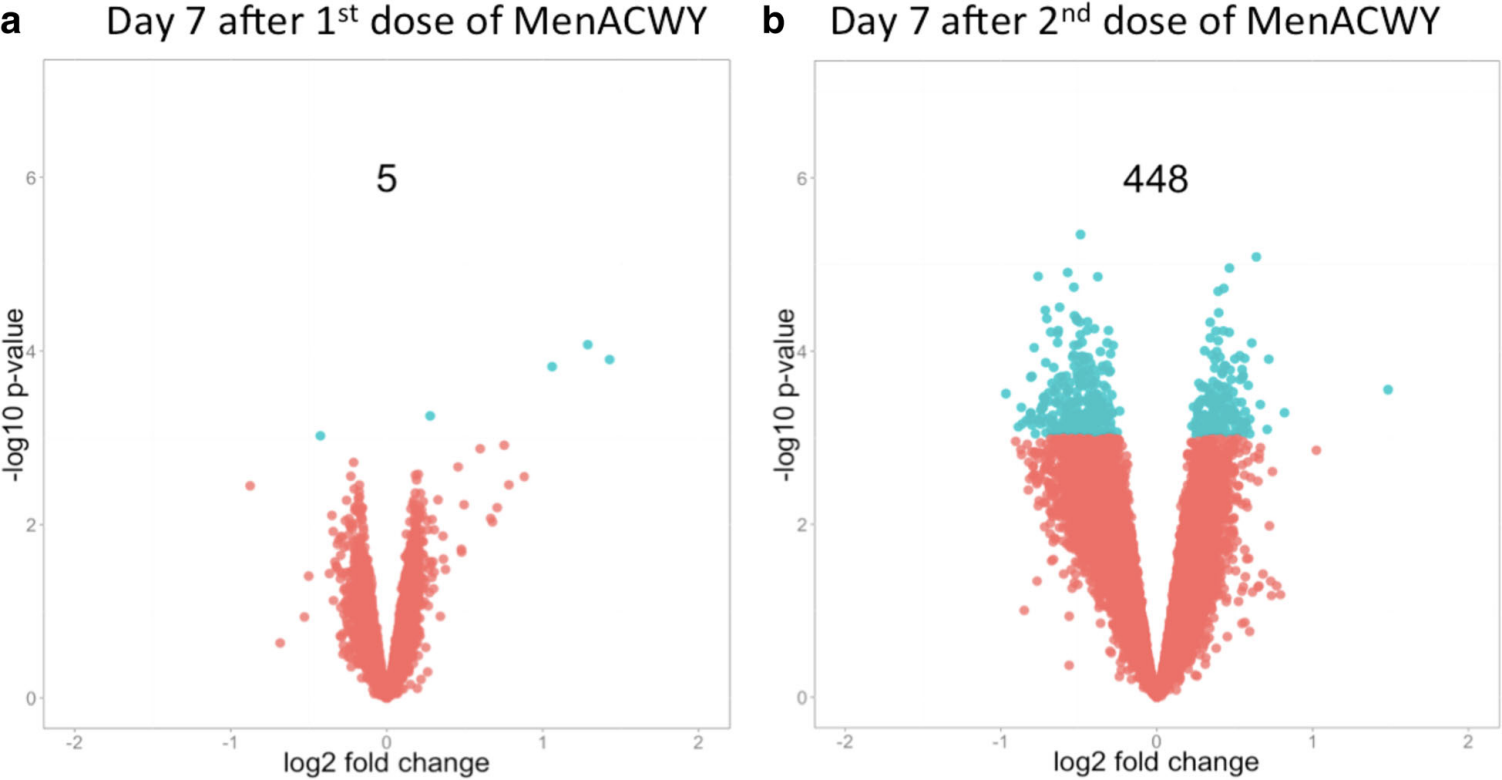
Differential transcript expression and associated *p* values following quadrivalent meningococcal vaccine (all groups combined). **a** Seven days after the initial dose of MenACWY. **b** Seven days after a second dose of MenACWY. Transcripts with a regression *p* value <0.001 are highlighted in *green*

The number of differentially expressed (*p* < 0.001) transcripts 7 days after the initial vaccination varied from 1 to 74, depending on the vaccine group (Additional file 1: Figure S1). We identified two transcripts (*IGLL1* and LOC642113) that were differentially expressed in more than one vaccine group, both of which were shared between groups 1 and 2 (Additional file 1: Figure S2). Conversely, significant overlap was seen between gene sets identified by GSEA, including up-regulation of gene sets enriched in IgD-negative B cells and induced 7 days after trivalent inactivated influenza vaccine (Additional file 1: Figure S2c). Moreover, hierarchical clustering showed more grouping by vaccination status (i.e. pre- versus post-vaccination) than vaccine group, which implies the former explains more of the variance observed in these data than the latter (Additional file 1: Figure S2). Given the hierarchical clustering and the significant overlap in gene regulation, as well as the small number of participants per group, we explored differentially transcript expression by combining all four vaccine groups (Fig. 3a). In this combined analysis, five transcripts were differentially expressed, three of which were annotated as antibody segments: LOC651751 (Ig kappa chain V-II region), LOC642113 (IGKV3D-20) and LOC649923 (Additional file 3: Table S2). The two other differentially expressed transcripts were a cyclin-dependent kinase inhibitor (*CDKN2D*) and a gene of unknown function (*LOC100131905*).

In addition to single-transcript-level analysis, gene set analysis can be evaluated, which increases statistical power by incorporating data from other relevant biological experiments. GSEA (http://www.broadinstitute.org/gsea/) was undertaken on the entire list of transcripts using the 1910 gene sets in the ‘c7: Immunological signature’ collection. Of note, 7 days post-MenACWY (combined group analysis), GSEA demonstrated the up-regulation of gene sets induced 7 days after trivalent inactivated influenza and yellow fever vaccines (Additional file 4: Table S3). Furthermore, GSEA at this time point also indicated the upregulation of gene sets associated with plasma cells (Additional file 4: Table S3). Analysis of blood transcriptional modules also showed enrichment of B-cell, plasmablast and immunoglobulin modules 7 days after vaccination (Additional file 1: Figure S3).

Next, we explored the difference in the mRNA transcripts induced by the two vaccines by comparing the fold-change in transcript expression 7 days (over baseline) following MenACWY-CRM (group 1) to that induced by MenACWY-PS (groups 2, 3 and 4); these vaccines differentially regulated a single transcript, *PRKAG2*, an adenosine monophosphate-activated protein kinase (Additional file 5: Table S4). GSEA revealed a number of immunological gene sets that were differentially regulated by these vaccines; in particular, a number of T cell-associated gene sets were comparatively upregulated in the MenACWY-CRM vaccinees (Additional file 6: Table S5).

### There is a correlation between transcriptomic and phenotypic plasma cell responses and vaccine immunogenicity for capsular group C meningococcal responses

The relationship between the frequency of total and plasma cell subsets and day 28 MenA- and MenC-specific SBA titres (all groups combined) are shown in Table 2. The most statistically significant correlation was seen between HLA-DR^hi^ CXCR3^+^ plasma cells, believed to represent newly generated plasmablasts, and MenC-specific SBA titres (Table 2) [17]. On the other hand, there was no evidence of correlation between these plasma cell populations and MenA-specific SBA titres. Furthermore, a statistically significant correlation was seen between day 7 MenC-specific ex vivo ASC frequencies and day 28 MenC-specific SBA titres, but not between MenA-specific ex vivo ASC frequencies and MenA-specific SBA titres (Additional file 1: Figure S6).

**Table 2:** Correlations between frequency of total and plasma cell subsets and day 28 MenA and MenC-specific serum bactericidal assay titres

Plasma cell subset	MenA SBA correlation (r)	MenA SBA correlation (*p* value)	MenC SBA correlation (r)	MenC SBA correlation (*p* value)
Total plasma cells	0.147	0.560	0.615	0.007
HLADR + CXCR3+	0.364	0.137	0.631	0.005
HLADR-CXCR3+	0.174	0.489	0.576	0.012
HLADR + CXCR3-	0.204	0.417	0.549	0.018
HLADR-CXCR3-	−0.015	0.954	0.504	0.033

*MenA* capsular group A meningococcus, *MenC* capsular group C meningococcus, *r* correlation coefficient

**Fig. 4:**
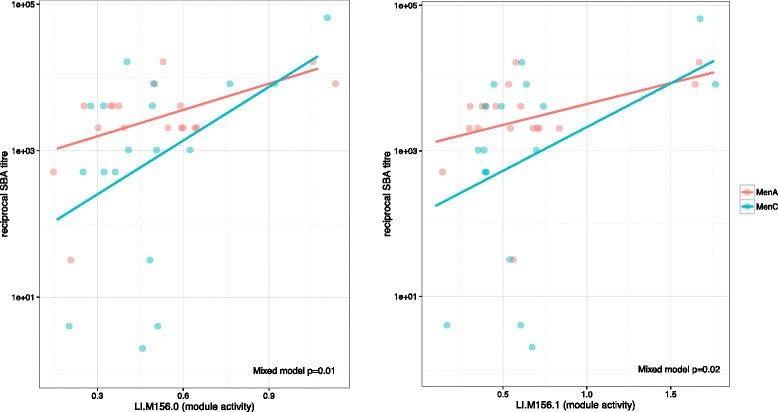
Correlation between B-cell and plasmablast module activity (mean of absolute fold changes) 7 days post-initial vaccine and day 28 MenA- and MenC-specific serum bactericidal assay (SBA) titres. Li.156.0, plasma cells and B cells, immunoglobulins; Li.156.1, plasma cells and immunoglobulins [14]

We next investigated whether the five transcripts that were differentially expressed following either MenACWY-PS or MenACWY-CRM correlated with subsequent measures of vaccine immunogenicity. The fold-change of two of these transcripts, corresponding to LOC651751 (Ig kappa chain V-II region) and LOC649923 (Ig gamma-2 chain C region), correlated with MenC-specific SBA titres 28 days post-vaccination (Additional file 1: Figure S7). Moreover, the fold-rise in LOC649923 and LOC642113 (IGKV3D-20) also correlated with MenC-specific cultured ASC ELISpots (Additional file 1: Figure S5). No statistically significant correlations were seen for any of these transcripts and MenA-specific SBA titres or cultured MenA-specific ASC ELISpot frequencies at 28 days (Additional file 1: Figures S4 and S5). However, mixed model analysis revealed a relationship between combined capsular group immunogenicity (MenA and MenC SBA titres) and activity of B-cell, plasmablast and immunoglobulin gene modules (Fig. 4).

To identify a parsimonious set of transcripts with fold changes that had a linear relationship with subsequent immunological phenotypes (Table 1), without imposing a threshold for differential expression, a regression shrinkage method (LASSO) was used [18, 19]. The fold change in a single transcript, *GNS*, at day 7 was significantly associated with MenC SBA 28 days after MenACWY when corrected for multiple testing (Additional file 7: Table S6). Moreover, the genes selected using the LASSO method were enriched for genes in pathways such as ‘cytokine signalling’ and ‘immune system’ (Additional file 8: Table S7). No gene fold changes were significantly associated with day 28 MenA-specific SBA titres when corrected for multiple testing.

### Disparate gene expression profiles observed 7 days after the second dose compared with those seen after the initial dose of MenACWY

A plot of mRNA transcript fold changes and accompanying *p* values 7 days after the second dose of quadrivalent meningococcal vaccine is shown in Fig. 3b. Seven days following the second vaccination (MenACWY-CRM), 448 transcripts were differentially expressed; this represents a notable enrichment of differentially expressed transcript (DETs) compared with 7 days following the initial dose of vaccine (Additional file 1: Figure S3a; Additional file 9: Table S8). Moreover, a number of these DETs were immune-related, including *TGFBR2*, which has an important role in immune regulation, binding TGF-β, subsequently triggering a signalling cascade that suppresses T- and B-cell proliferation and effector function [20, 21, 22]. Contemporaneously, *NFKB2*, a central activator of genes involved in inflammation and immune function, was upregulated [23]. B cell-specific transcripts such as *FCRLA* and *BANK1* were downregulated; conversely, *FOXP1*, a transcription factor essential for B-cell development and germinal centre regulation, was upregulated, as was *SIVA1*, which binds to the tail of *CD27* (present on a subset of T and B cells), inducing apoptosis [24].

**Fig. 5:**
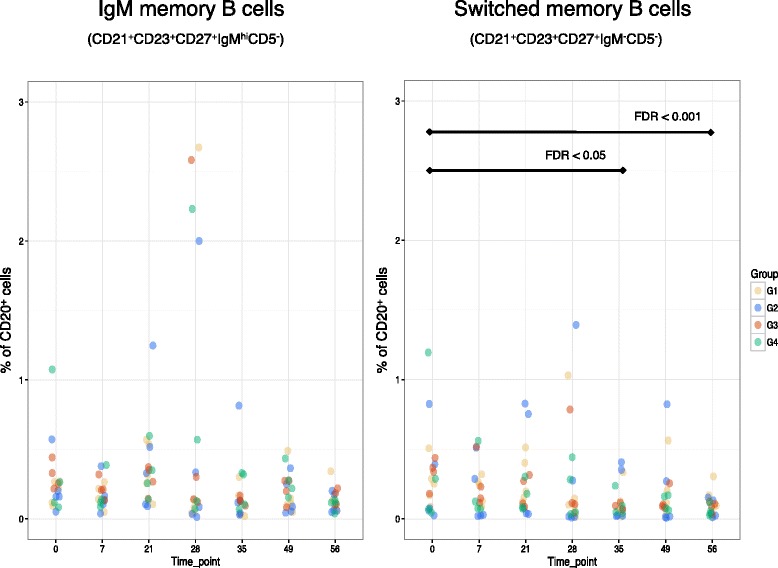
Flow cytometric analysis of IgM memory (*left*) and switched memory (*right*) B cell populations, shown as the percentage of total B cells (CD20^+^) at various study time points (i.e. initial vaccine at day 0 followed by an additional dose at day 28). *FDR* false discovery rate

GSEA of mRNA transcript profiles 7 days after the second dose of vaccine showed the regulation of several gene sets, including the downregulation of a ‘switch memory B cells compared with IgM memory B cells’ gene set (Additional file 10: Table S9). These data were mirrored by flow cytometric data showing a relative decline in switched memory B cells 7 days after the second dose of vaccine (Fig. 5).

Next, we assessed transcriptional responses following the second dose of vaccine in those who previously received MenACWY-CRM compared with those who received a prior dose of MenACWY-PS, finding 32 transcripts that were differentially regulated between these groups (group 1 versus groups 2, 3 and 4; Additional file 11: Table S10). Moreover, GSEA of these data showed the enrichment of a number of gene sets; of particular interest, the downregulation of the ‘switched memory B cells compared with IgM memory B cells’ gene set was found to be driven by those who previously received a dose of MenACWY-PS (Additional file 12: Table S11). When the data were subsetted by initial vaccine the ‘switched memory B cells compared with IgM memory B cells’ gene set was exclusively downregulated in the MenACWY-PS cohort, with a greater enrichment score than for the combined analysis (data not shown).

### The correlation between the transcriptomic profile induced at 7 days and vaccine immunogenicity is reproduced in an independent dataset

**Fig. 6:**
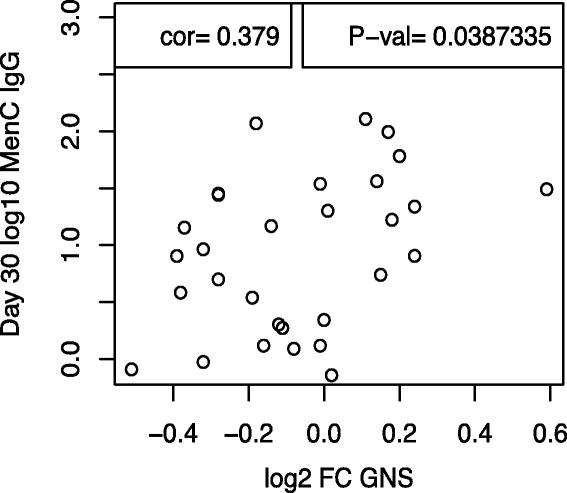
Correlation between the log_2_ fold change from day 0 to day 7 in *GNS* and MenC-specific IgG concentrations 30 days after MenACWY vaccine. The Pearson product–moment correlation coefficient (*cor*) and accompanying *p* value are displayed

We then explored whether the expression of genes identified in our data set could be validated in an independent cohort; for this purpose we utilised a publicly available dataset, acquired from the genomics data repository Gene Expression Omnibus (http://www.ncbi.nlm.nih.gov/geo/). This dataset comprised adults vaccinated with either MenACWY-PS (*n* = 13) or MenACWY-CRM (*n* = 17), as described by Li et al. [14]. That study also described upregulation of transcripts annotated as ‘immunoglobulin’ 7 days post-MenACWY [14]. We assessed whether antibody segments that associated with MenC-specific SBA in our dataset also correlated with immunological data available in the Li et al. study. However, as different microarrays were used in the two studies it was not possible to directly compare transcripts; instead, comparisons were made at the level of the respective genes (i.e. *IGKC* and *IGHG2*). While we did not replicate correlations between these genes and MenC SBA titres, a statistically significant correlation was seen with anti-diphtheria toxoid antibody concentrations (Additional file 1: Figure S9). Next, we evaluated the relationship between *GNS* expression and MenC responses in the Li et al. dataset, finding a statistically significant correlation between the expression of this gene and day 30 MenC IgG concentrations (Fig. 6).

### *XBP1* upregulated 7 days after either plain polysaccharide or conjugate MenACWY vaccine

Interestingly in the Li et al. dataset, 440 transcripts were differentially regulated 7 days after MenACWY-CRM compared with MenACWY-PS; amongst the most differentially expressed transcripts were genes involved in responses to unfolded protein pathway, including *XBP1* (Additional file 13: Table S12). Moreover, GSEA showed significant enrichment of gene sets associated with ‘unfolded protein response’ and ‘activation of chaperone genes by XBP1s’ 7 days after MenACWY-CRM compared with the equivalent time point following MenACWY-PS (Additional file 14: Table S13). Conversely, the microarray data in our study did not show differences in expression of *XBP1* between MenACWY-CRM and MenACWY-PS vaccinees; however, scrutiny of the *XBP1* probe sequences on this microarray platform implied that these would not be able to distinguish between the unspliced (XBP1u) and spliced (XBP1s) isoforms of this gene, the former being constitutively expressed and the latter being the potent transcription factor implicated in plasma cell differentiation [25]. To address this query, we conducted quantitative PCR using primers specific for the spliced and unspliced isoforms of *XBP1*, finding upregulation of XBP1s but not XBP1u 7 days after an initial dose of either MenACWY-CRM or MenACWY-PS vaccine (Additional file 1: Figure S11). However, we did not demonstrate a statistically significant difference in the induction of XBP1s between those who received the conjugated or plain polysaccharide vaccine (*p* = 0.22).

## Discussion

In this report we describe novel and reproducible transcriptional correlates of vaccine immunogenicity. Seven days after a dose of MenACWY vaccine, plasma cell responses, measured both phenotypically and at the transcriptomic level, correlated with subsequent MenC-specific antibody titres. Notably, 7 days after an additional dose of MenACWY vaccine, given one month later, highly disparate responses were observed compared with the initial dose. Intriguingly, considerable differences in transcriptional responses after the second dose of vaccine were seen in those who received a prior dose of MenACWY-PS compared with those who previously received a MenACWY-CRM vaccine, with downregulation of a ‘switched memory B cell’ gene set exclusively observed in those who previously received the plain polysaccharide vaccine.

The transcriptomic profile 7 days after the initial dose of MenACWY vaccine was characterised by differential expression of transcripts annotated as antibody segments; fold-changes in *IGKC* and *IGHG2* genes correlated with MenC-specific SBA titres and memory B cell ELISpots 28 days post-vaccination. *IGHG2* encodes the constant region of the heavy chain of IgG2, which is the predominant IgG subclass directed to polysaccharide antigens (including those in protein-conjugated meningococcal vaccines) in older children and adults [26, 27, 28]. It is possible to infer from the correlation between the expression of *IGHG2* and MenC-SBA titres that this gene is a marker of a population of MenC-specific plasma cells, secreting high avidity complement-fixing IgG2. While IgG2 is not as efficient at fixing complement as IgG1, it can activate the classic pathway at high epitope density and when antibody is at equivalence or in excess [29]. We were also able to describe statistically significant relationships between the activity of B-cell, plasmablast and immunoglobulin gene modules assessed 7 days after vaccination and later measures of vaccine immunogenicity (28 days post-vaccination); this is consistent with previous reports that have correlated these modules with responses to other vaccinations such as pneumococcal polysaccharide vaccine and trivalent-inactivated influenza vaccine [14, 30]. Further description of the B-cell receptor transcript repertoire in this study is described elsewhere [31].

While we observed a correlation between day 7 MenC-specific ex vivo ASC frequencies and day 28 MenC-specific SBA titres, we did not see a statistically significant correlation between MenA-specific ex vivo ASC frequencies and MenA-specific SBA titres. A possible explanation for this disparity could be lack of statistical power, given the modest number of participants assessed; alternatively, it may be due to intrinsic differences between the ex vivo ELISpot, which measures total MenA-specific ASCs, and the MenA-specific SBA, which measures a subset of antibodies with functional activity (bactericidal).

To further investigate predictors of vaccine immunogenicity, we identified a minimal set of transcripts that independently predicted post-vaccination SBA titres using a shrinkage variable selection algorithm (LASSO); the resultant gene set was enriched for transcripts involved in pathways such as ‘cytokine signalling’ and the ‘immune system’. A statistically significant association was seen between the fold rise in *GNS* at day 7 and day 28 MenC-specific SBA titres. Additionally, a correlation between day 7 *GNS* expression and day 30 MenC IgG concentrations was also observed in the Li et al. cohort. Glucosamine (N-acetyl)-6-sulfatase (GNS) is a lysosomal enzyme found in all cells and has been shown to be perturbed following yellow fever and live-attenuated influenza vaccine [32, 33]. Deficiencies in *GNS* result in the lysosomal storage disorder mucopolysaccharidosis type IIID (MPS IIID), a condition predominantly characterised by severe neurological manifestations; however, this condition is also associated with recurrent ear and upper respiratory infections [34]. These data show a relationship between expression of *GNS* and antibody responses against polysaccharide-encapsulated bacteria and, together with the observations in MPS IIID, indicate a potential role for this gene in controlling infection caused by encapsulated bacteria [34].

Analysis of day 7 data following the second dose of vaccine appeared highly disparate from those observed after the initial dose, with a notable enrichment of DETs. B cell-specific transcripts, such as *FCRLA* and *BANK1*, were downregulated at this time point; conversely, *FOXP1*, which has been shown to repress plasma cell differentiation, was upregulated [35]. Pro-apoptotic *SIVA1* was also upregulated, which binds to the cytoplasmic tail of *CD27*, expressed by a subpopulation of T and B cells, including plasma cells [24]. At this time point we also observed the downregulation of *TGFBR2*, which is essential for the normal maintenance of conventional B cells [20]. Concurrently, *NFKB2* (subunit of NF-kB), which is induced downstream of several pathways, including signalling via the B-cell receptor (BCR), was upregulated [36]. While NF-kB is often regarded as a prototypical pro-inflammatory factor, it also has an important role in limiting inflammatory responses, for example by promoting activation-induced cell death of T and B cells [37]. It is possible to postulate these data may partly reflect immunoregulatory processes induced by repeated exposure to recently encountered (or persistent) antigens.

GSEA of gene profiles 7 days after the second dose of vaccine indicated the downregulation of the ‘switched memory B cells compared with IgM memory B cells’ gene set; however, this observation was exclusively driven by individuals who previously received the plain polysaccharide vaccine. Flow cytometric analysis implied that this observation was due to a decrease in switched memory B cells rather than an increase in IgM memory B cells. While previous data have shown the depletion of antigen-specific switched memory B cells following polysaccharide vaccines, remarkably, we witnessed a reduction in the total frequency of these cells [6, 38]. Murine studies have shown polysaccharide vaccines deplete antigen-specific memory B cells by inducing apoptosis, which has been proposed as the mechanism underlying hyporesponsiveness following these vaccines [38]. However, factors involved in isotype switching, such as TACI and BAFFR, have been shown to be downregulated by B cells following administration of capsular group C meningococcal polysaccharide vaccine, resulting in less BAFF- and APRIL-induced IgG secretion [39]. The aforementioned study proposed this as an additional mechanism, independent of B-cell receptor specificity, by which polysaccharide vaccines induce suppression of B-cell responses [39]. Importantly, the downregulation of the ‘switched memory B cells compared with IgM memory B cells’ gene set was solely observed in individuals who previously received MenACWY-PS, which is consistent with the notion that hyporesponsiveness is not a feature of conjugate vaccines [40]. Nevertheless, these data imply that an additional dose of MenACWY-CRM one month after MenACWY-PS does not mitigate the depletion of switched memory B cells, and therefore theoretically hyporesponsiveness.

As the number of participants involved in this study was modest, some of the deductions made from this work merit further confirmatory investigation. To strengthen some of our conclusions we reproduced some of our finding in a publicly available dataset. Interestingly, in contrast to the Li et al. study, we did not observe differences in the expression of *XBP1* between MenACWY-CRM and MenACWY-PS vaccinees; rather, we found the functional transcript (XBP1s) to be upregulated 7 days after an initial dose of either vaccine. XBP-1 is a transcription factor that is a key regulator of major histocompatibility complex class II expression on B cells and is critical to the function of highly secretory cells such as plasma cells [25, 41]. XBP1 is critical for normal plasma cell secretory function, which would logically explain its upregulation coinciding with the peak plasma cell frequency in peripheral blood following vaccination [25, 42]. Interestingly, *XBP1* did not appear to be upregulated 7 days after plain polysaccharide vaccine in the Li et al. dataset, which corresponded with modest plasma cell responses to this vaccine. A possible explanation for the disparities between our *XBP1* findings and those described by Li et al. may be differing polysaccharide-specific plasma cell kinetics due to dissimilarities in the pre-existing immune status of the study participants. The appearance of plasma cells in peripheral blood has been shown to differ between primary and secondary immune responses, peaking at 10 and 7 days, respectively [43]. While Li et al. observed that the vast majority of plasma cells induced by the protein-conjugate meningococcal vaccine were carrier protein-specific and that the plain polysaccharide vaccine induced few plasma cells 7 days post-vaccination, our data indicated comparable plasma cell responses between these vaccines [14]. It may be the case that historical differences in meningococcal nasopharyngeal carriage incidence between these populations underlie these observations. Importantly, carriage varies by country; for example, the epidemic of MenC disease observed in the UK in the late 1990s was not seen in the US [44]. Furthermore, the UK participants were older (30–70 years of age, median 55 years, interquartile range 44–59 years) than the US participants (18–45 years of age), and therefore have an increased risk of having previously carried as a function of time [45]. In addition, these disparities may reflect intrinsic differences between the vaccines administered in these studies (here, Menveo® (Novartis Vaccines) and/or ACWYVax® (GlaxoSmithKline); Li et al., Menomune® (Sanofi Pasteur) or Menactra® (Sanofi Pasteur)).

In this study we observed similar MenA- and MenC-specific SBA titres following a single dose of both MenACWY-PS and MenACWY-CRM, which is consistent with previous immunogenicity data in adults [46, 47]. No further rise in SBA titres was seen following the additional dose of vaccine; this is not surprising as polysaccharide vaccines do not induce immunology memory, and a minimal interval of ~4 months is required for effective booster responses [48]. Interestingly, we demonstrated inferior MenC-specific ex vivo ASC frequencies following the second dose of vaccine in those who previously received the plain polysaccharide vaccine, compared with those who received the protein-conjugated vaccine; these data are consistent with polysaccharide vaccine-induced hyporesponsiveness. While this was not seen with regards to MenA-specific ex vivo ASCs, this may reflect lack of power to demonstrate statistical significance.

## Conclusions

We describe distinct gene signatures that appear to be associated with the production of high-avidity antibody and control of B-cell responses after repeated doses of meningococcal vaccines. We demonstrate the downregulation of a gene set associated with switched memory B cells that was unique to those who received an initial dose of plain polysaccharide vaccine, which may represent an early molecular signal of hyporesponsiveness induced by this vaccine.

## Additional files

Additional file 1: A PDF containing all supplementary figures [49]. (PDF 1469 kb)
Additional file 2: Table S1. Study demography. (XLS 38 kb)
Additional file 3: Table S2. Differentially transcript expression 7 days after an initial dose of MenACWY. (XLS 2176 kb)
Additional file 4: Table S3. Gene set enrichment analysis of gene expression data 7 days after an initial dose of MenACWY vaccine. (XLS 36 kb)
Additional file 5: Table S4. Comparison of transcript fold changes 7 days after vaccination with MenACWY-CRM or MenACWY-PS. (XLS 2331 kb)
Additional file 6: Table S5. Gene set enrichment analysis of gene expression data 7 days after MenACWY-CRM compared to MenACWY-PS vaccine. (XLS 41 kb)
Additional file 7: Table S6. Significance testing of the transcripts at day 7 post-vaccination that were selected as predictor variables of day 28 MenC-specific SBA by the LASSO model, using the covTest R package. (XLS 48 kb)
Additional file 8: Table S7. Pathway analysis for the transcripts at day 7 post-vaccination that were selected as predictor variables of day 28 MenC-specific SBA by the LASSO model. (XLS 35 kb)
Additional file 9: Table S8. Differential transcript expression 7 days after a booster dose of MenACWY. (XLS 2429 kb)
Additional file 10: Table S9. Gene set enrichment analysis of gene expression data 7 days after booster dose of MenACWY. (XLS 118 kb)
Additional file 11: Table S10. Comparison of transcript fold changes 7 days after a second dose of MenACWY in those who previously received a dose of MenACWY-CRM or MenACWY-PS. (XLS 2367 kb)
Additional file 12: Table S11. Gene set enrichment analysis of gene expression data 7 days after a second dose of MenACWY-CRM in those primed with MenACWY-CRM compared to those who previously received a dose of MenACWY-PS. (XLS 100 kb)
Additional file 13: Table S12. Genes differentially expressed 7 days after conjugate vaccine compared with polysaccharide vaccine in the Li et al. dataset. (XLS 5370 kb)
Additional file 14: Table S13. Gene set enrichment analysis of gene expression data 7 days after MenACWY-CRM compared with MenACWY-PS vaccine in the Li et al. dataset. (XLS 12 kb)

## Abbreviations

ASC: Antibody-secreting cell
BSA: Bovine serum albumin
CD: Cluster of differentiation
CRM: Cross-reactive material
DET: Differentially expressed transcript
GMT: Geometric mean titre
GSEA: Gene-set enrichment analysis
Ig: Immunoglobulin
MenA: Group A meningococcus
MenACWY: Group A,C,W,Y meningococci
MenC: Group C meningococcus
MPS IIID: Mucopolysaccharidosis type IIID
NIBSC: National Institute for Biological Standards and Control
PBMC: Peripheral blood mononuclear cell
PBS: Phosphate-buffered saline
PI: Propidium iodide
PS: Polysaccharide
SBA: Serum bactericidal assay
