# Supplementary material for: High-dimensional assessment of B-cell responses to quadrivalent meningococcal conjugate and plain polysaccharide vaccine
Source: Genome Med. 2017 Jan 30;9:11. doi: 10.1186/s13073-017-0400-x (PMC5282650; doi:10.1186/s13073-017-0400-x)
Supplement: Additional file 1: — A PDF containing all supplementary figures [49]. (PDF 1469 kb) [file 13073_2017_400_MOESM1_ESM.pdf]

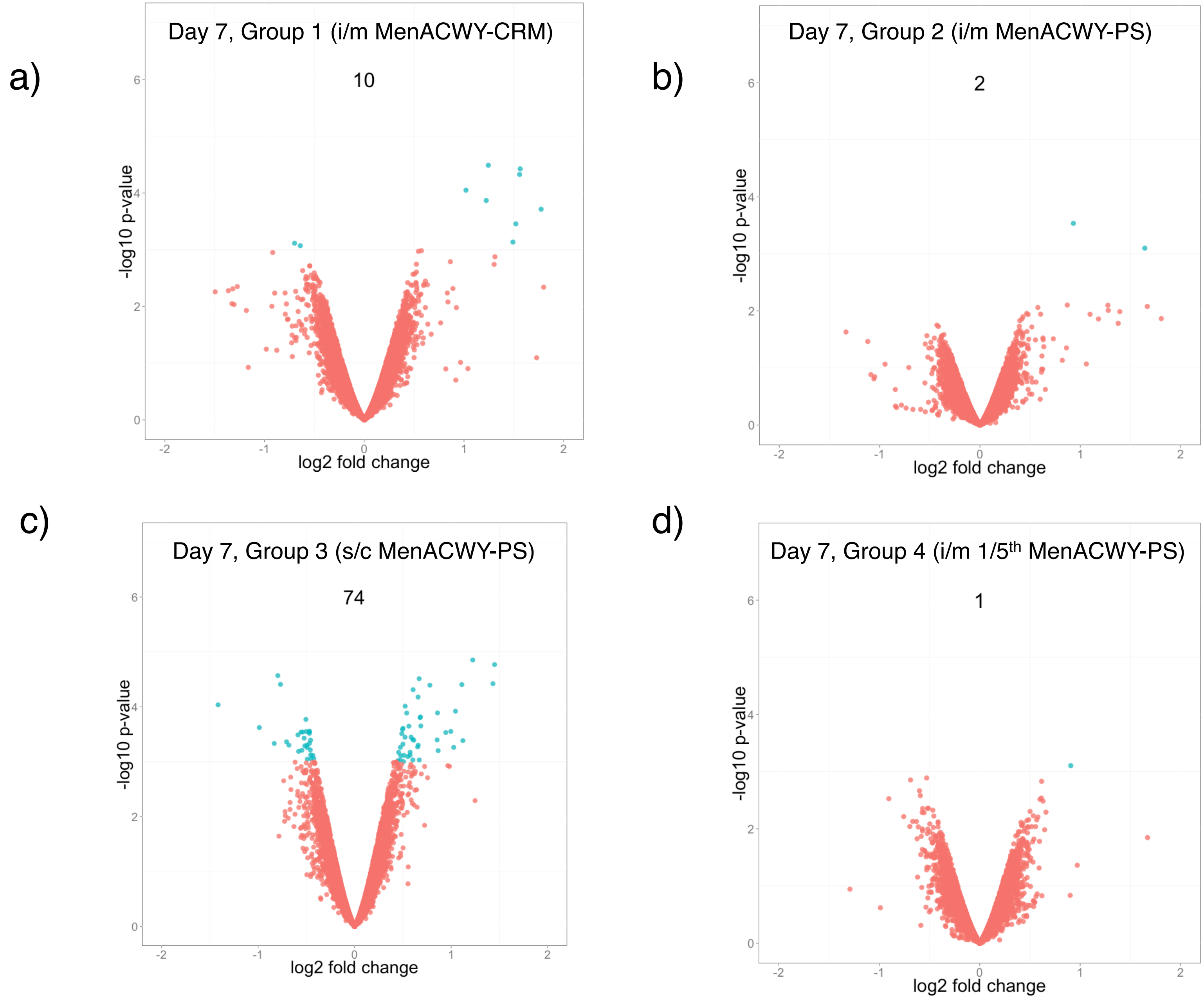


**Supplementary figure 1:** Volcano plot of fold-change and regression associated p-value 7 days after first vaccination; a) Group1 (intramuscular MenACWY-CRM), b) Group 2 (intramuscular MenACWY-PS), c) Group 3 (subcutaneous MenACWY-PS) and d) Group 4 (1/5th dose intramuscular MenACWY-PS).

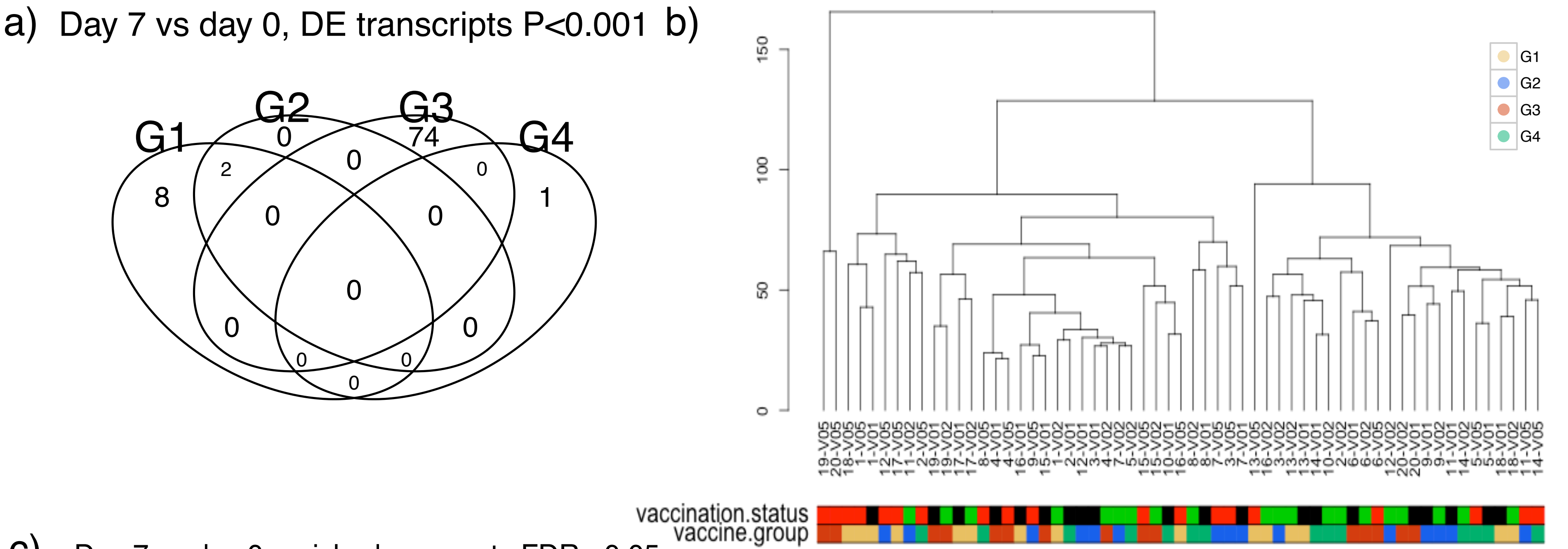


c) Day 7 vs day 0 enriched gene sets FDR <0.05

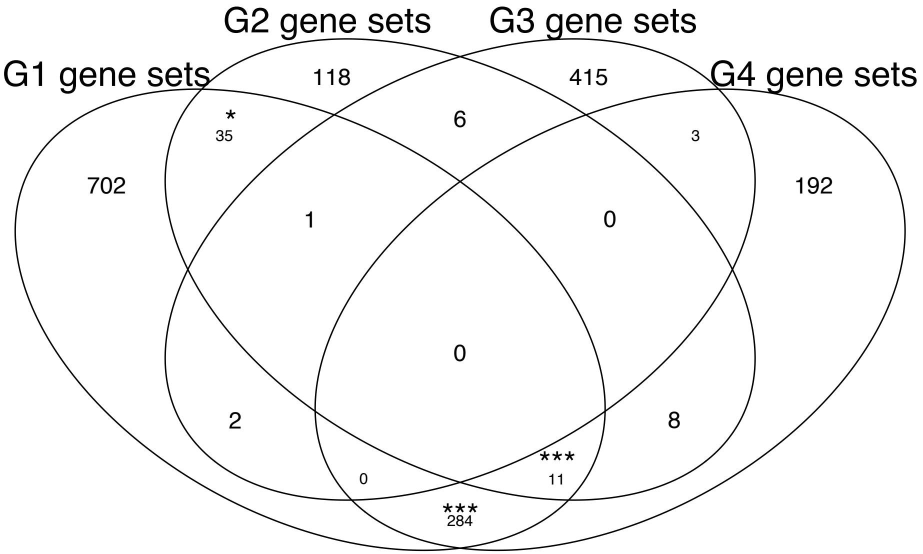


| Groups | Gene Set | Direction |
|---|---|---|
| G1,2,4 | GSE29614_DAY3_VS_DAY7_TIV_FLU_VACCINE_PBMC_DN | Positively enriched |
| | GSE29614_CTRL_VS_DAY7_TIV_FLU_VACCINE_PBMC_DN | Positively enriched |
| | GSE29164_CD8_TCELL_VS_CD8_TCELL_AND_IL12_TREATED_MELANOMA_DAY7_DN | Positively enriched |
| | GSE29618_PDC_VS_MDC_DAY7_FLU_VACCINE_UP | Positively enriched |
| | GSE10239_NAIVE_VS_DAY4.5_EFF_CD8_TCELL_DN | Positively enriched |
| | GSE12366_GC_VS_NAIVE_BCELL_UP | Positively enriched |
| | GSE22886_NEUTROPHIL_VS_DC_DN | Positively enriched |
| | GSE22886_NAIVE_CD4_TCELL_VS_48H_ACT_TH1_DN | Positively enriched |
| | GSE12845_IGD_POS_VS_NEG_BLOOD_BCELL_DN | Positively enriched |
| | GSE9946_IMMATURE_VS_LISTERIA_INF_MATURE_DC_DN | Positively enriched |
| | GSE13547_CTRL_VS_ANTI_IGM_STIM_BCELL_12H_DN | Positively enriched |
| | | |
| G1,2,3 | GSE13411_PLASMA_CELL_VS_MEMORY_BCELL_UP | Positively enriched |

**Supplementary figure 2:** a) and b) venn diagrams of differentially expressed gene between groups at p <0.001 and p<0.01, respectively, c) Venn diagram of significantly enriched gene sets (consistent direction of regulation) from gene set enrichment analysis and table of gene sets identified in all 4 of the vaccine groups. P-values were derived using the "SuperExactTest" R package (Wang et al., 2015), which was only performed for intersections between ≥3 groups. # = p-value = 0.05, * = p-value <0.05, ** =p-value <0.01, and *** = p-value <0.001.

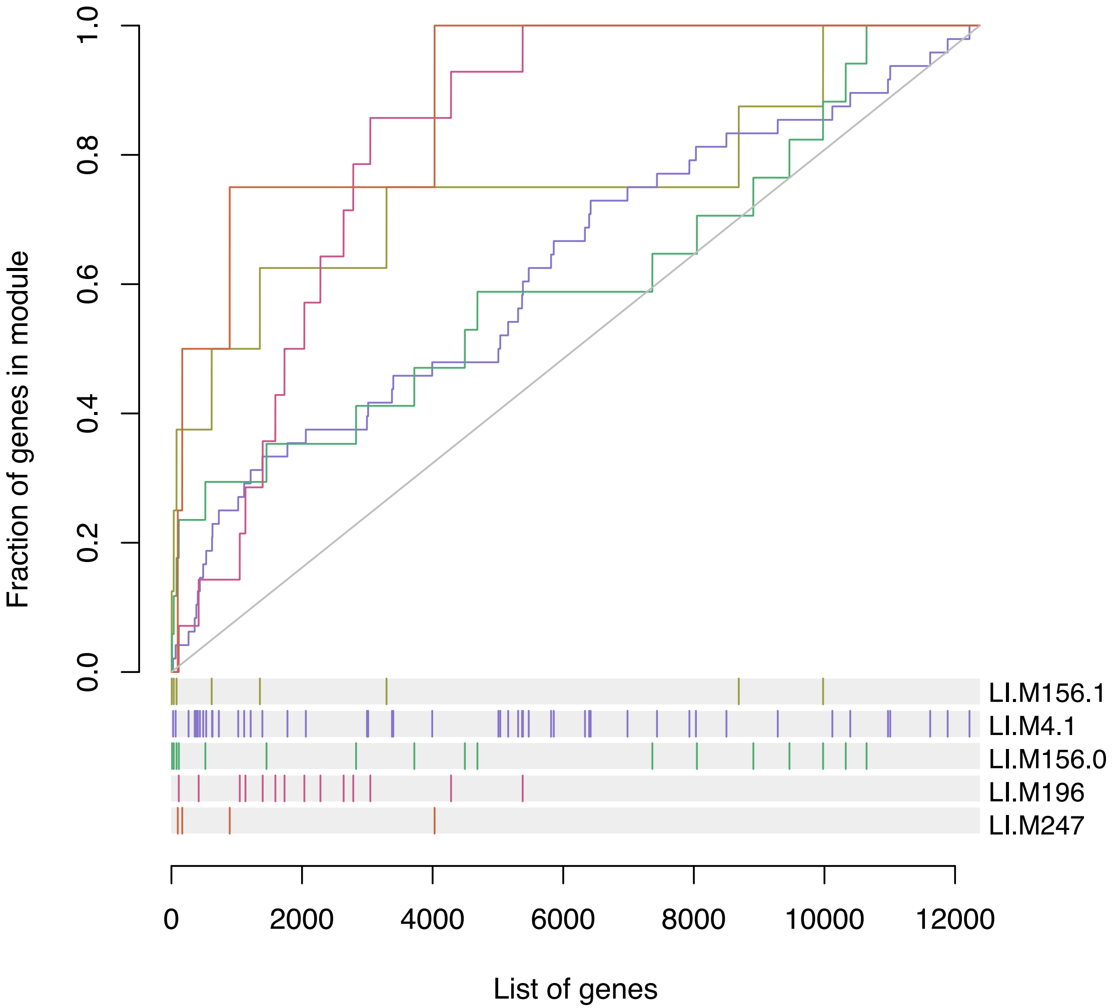


| ID | Title | cerno | AUC | cES | P.Value | adj.P.Val |
|---|---|---|---|---|---|---|
| LI.M156.1 | plasma cells, immunoglobulins | 51.62686 | 0.757326 | 3.226679 | 1.26E-05 | 0.004353 |
| LI.M4.1 | cell cycle (I) | 153.5919 | 0.62314 | 1.599915 | 0.000173 | 0.029913 |
| LI.M156.0 | plasma cells & B cells, immunoglobulins | 69.54371 | 0.607302 | 2.045403 | 0.000308 | 0.035561 |
| LI.M196 | platelet activation - actin binding | 57.94998 | 0.827994 | 2.069642 | 0.000739 | 0.063947 |
| LI.M247 | enriched in nuclear pore complex interacting protei | 25.80393 | 0.895414 | 3.225491 | 0.001135 | 0.075221 |

**Supplementary figure 3:** Receiver-operator characteristic (ROC) curve, of the distribution of the module genes sorted by t-statistic, using “tmod” R package (Weiner, 2016).

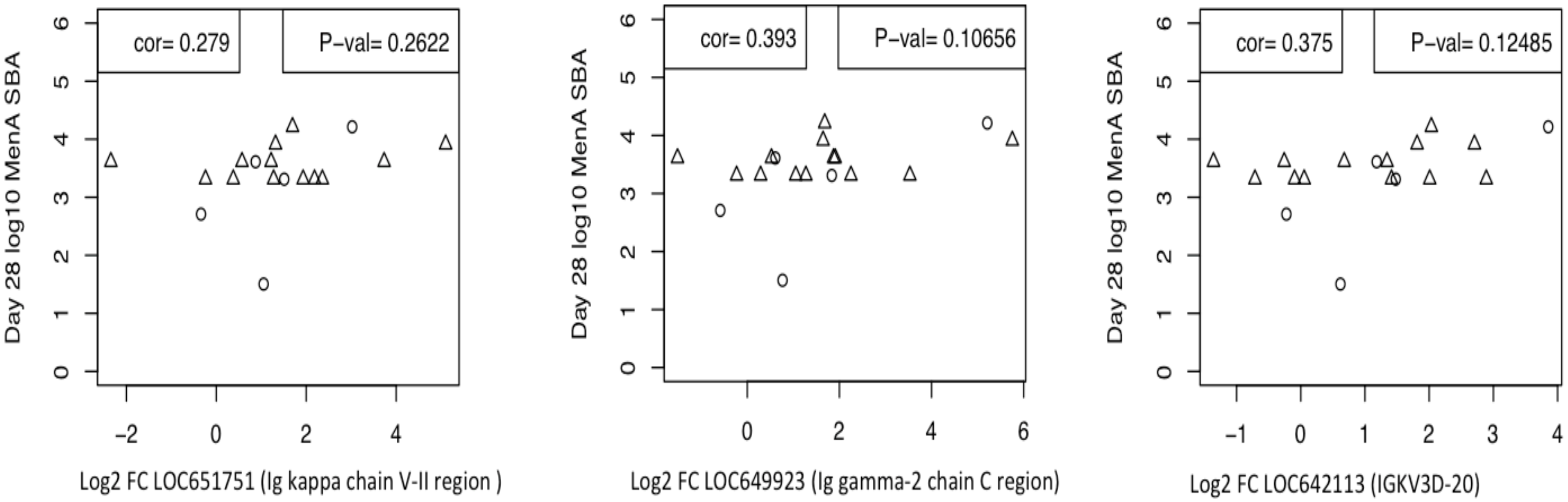


**Supplementary figure 4:** Correlation between the $\log_2$ fold-change from day0 to day 7 in three differentially expressed ($p<0.001$) 'immunoglobulin' mRNAs and MenA-specific SBA titres 28 days after MenACWY vaccine. Triangles represent individuals who received MenACWY-PS and circles received MenACWY-CRM. Pearson product-moment correlation coefficient (cor) and accompanying p-value are displayed.

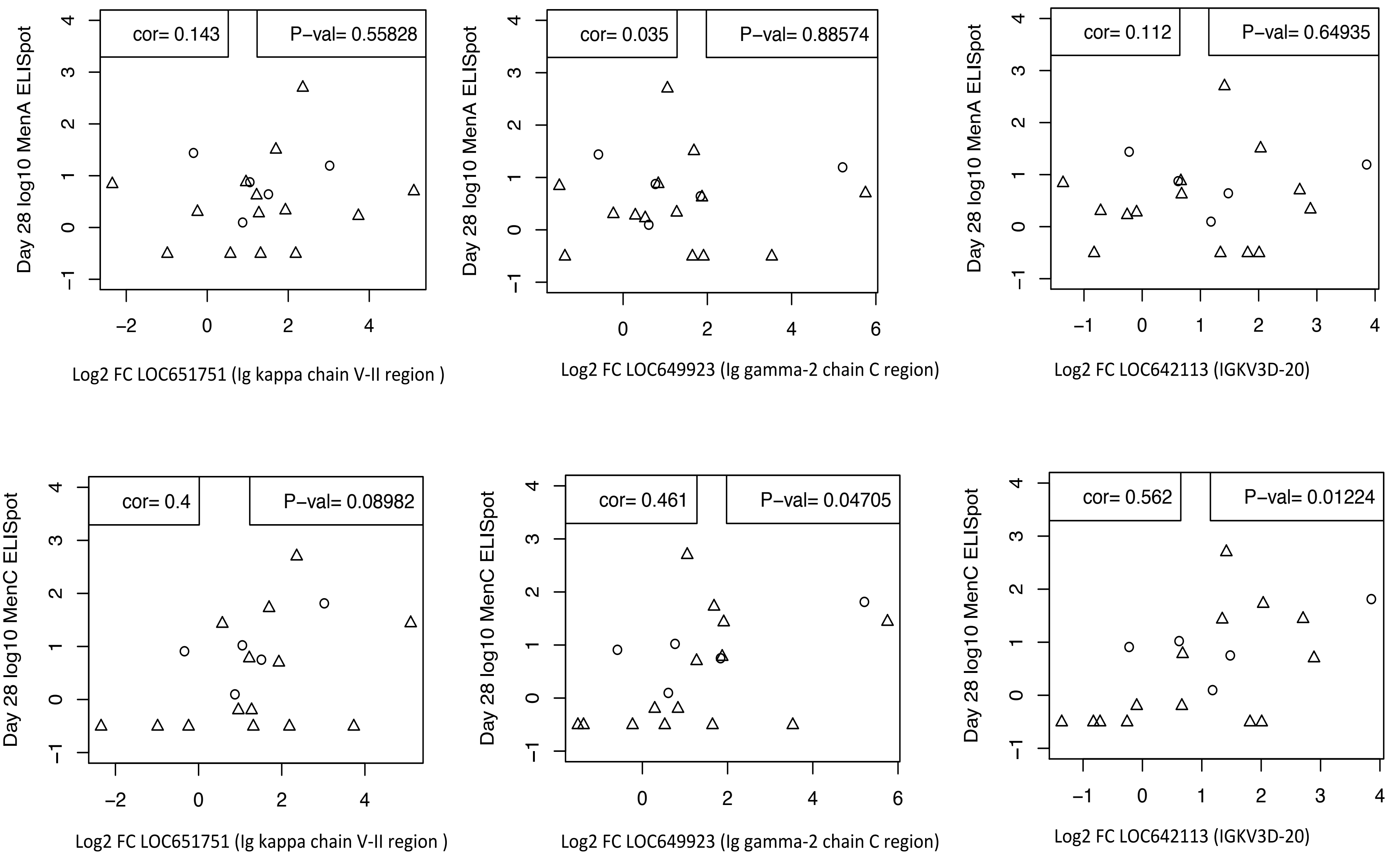


**Supplementary figure 5:** Correlation between the $\log_2$ fold-change from day0 to day 7 in three differentially expressed ($p<0.001$) ‘immunoglobulin’ mRNAs and ELISpots 28 days after MenACWY vaccine. Top row MenA, bottom row MenC. Pearson product-moment correlation coefficient (cor) and accompanying p-value are displayed.

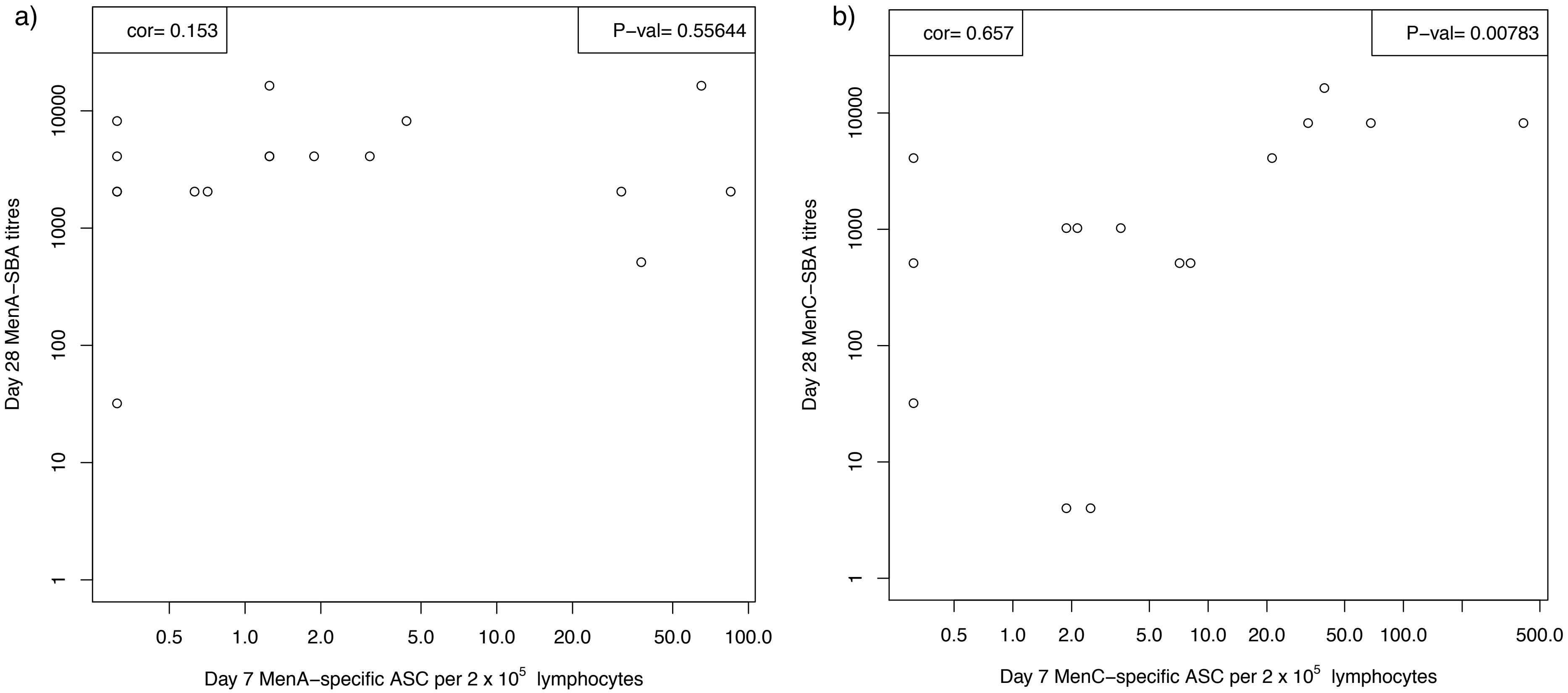


**Supplementary figure 6:** a) Relationship between day 7 ex vivo MenA-specific antibody-secreting cells (ASCs) and day 28 MenA-specific serum bactericidal assay (SBA) titres. b) Relationship between day 7 ex vivo MenC-specific antibody-secreting cells (ASCs) and day 28 MenC-specific serum bactericidal assay (SBA) titres. Pearson product-moment correlation coefficient (cor) and accompanying p-value are displayed.

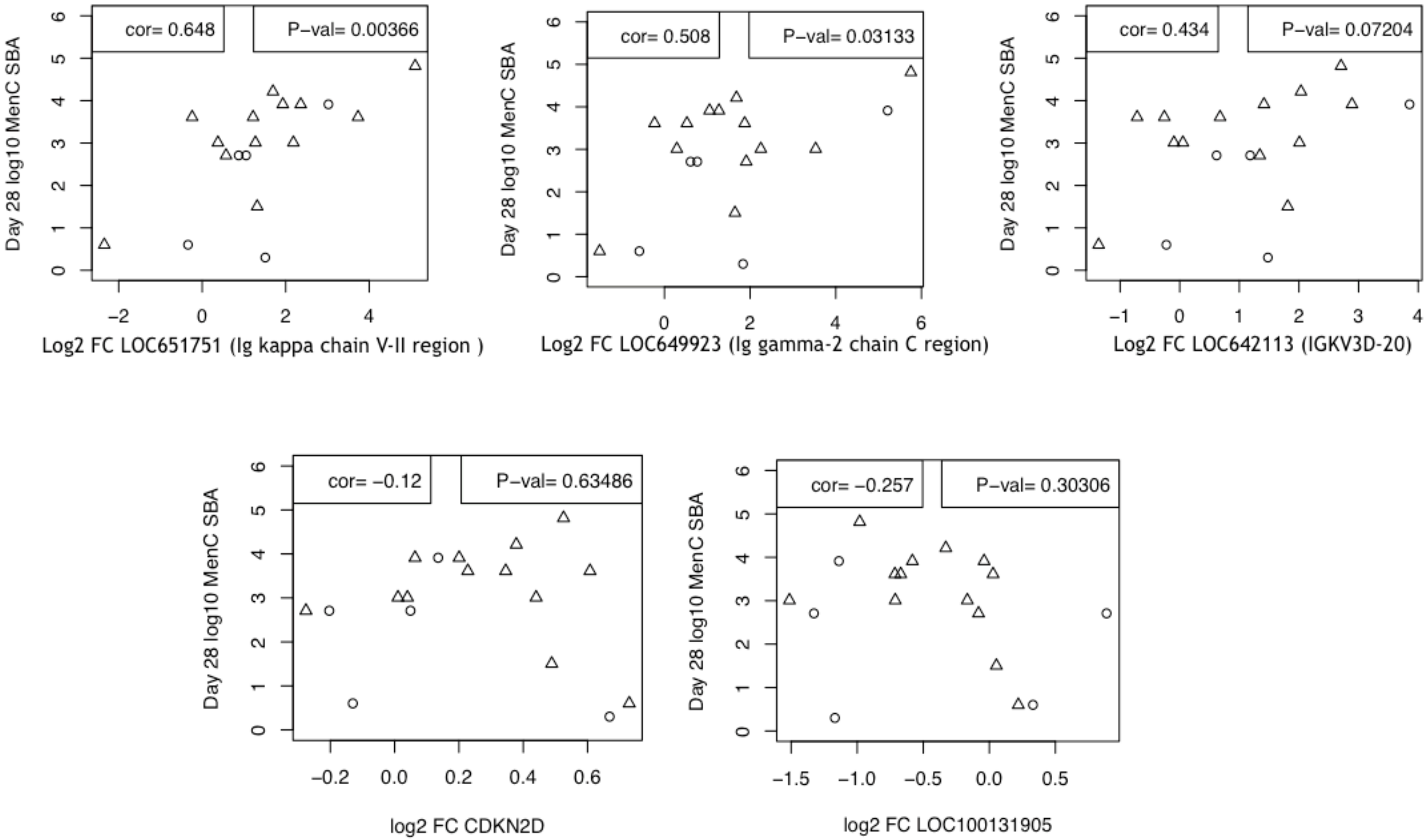


**Supplementary figure 7:** Correlation between the $\log_2$ fold-change from day0 to day 7 in five, differentially expressed (day7, p<0.001), mRNA transcripts and MenC-specific SBA titres 28 days after MenACWY vaccine. Individuals who received MenACWY-CRM are depicted with circles and those who received MenACWY-PS with triangles. Pearson product-moment correlation coefficient (cor) and accompanying p-value are displayed.

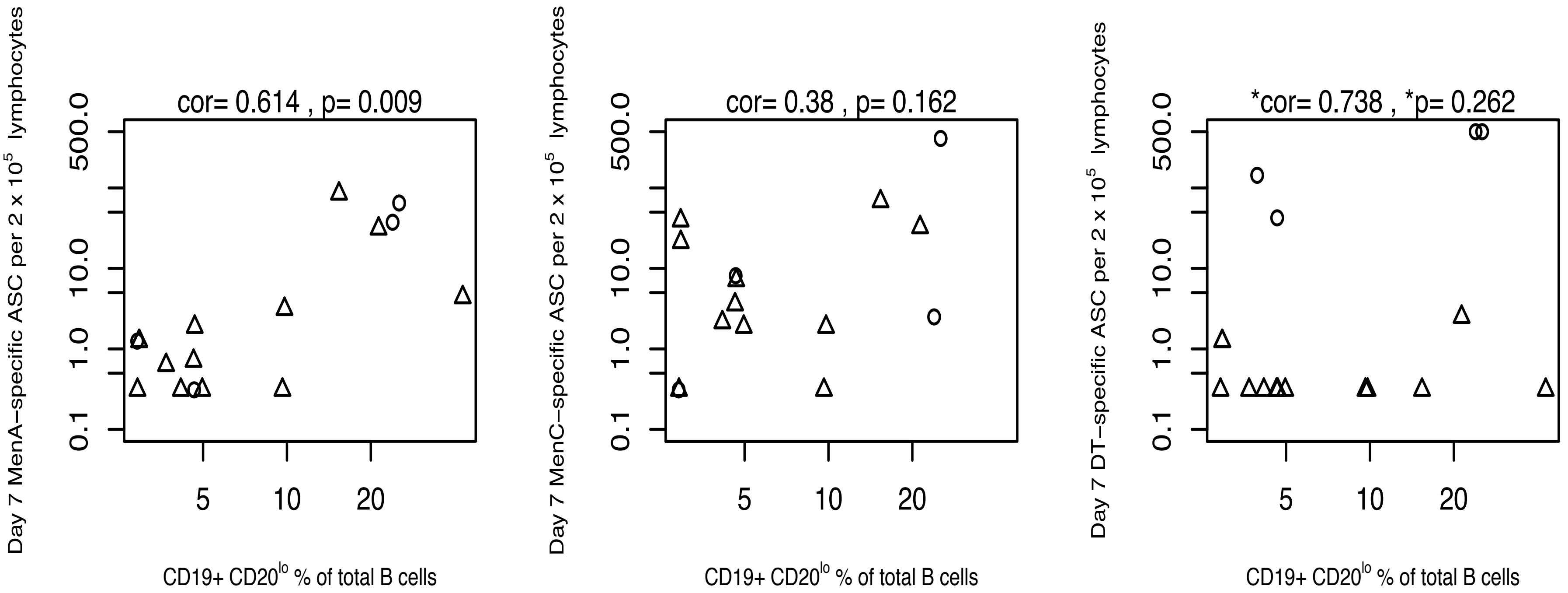


**Supplementary figure 8:** Correlation between the antigen-specific ex vivo ELISpot counts and total plasma cell measured 7 days post-vaccination, by FACs. Spearman's rank correlation coefficient (cor) and accompanying p-value are displayed. Individuals who received MenACWY-CRM are depicted with circles and those who received MenACWY-PS with triangles. * denotes only individuals who received DT containing vaccine (circles) were included in this analysis.

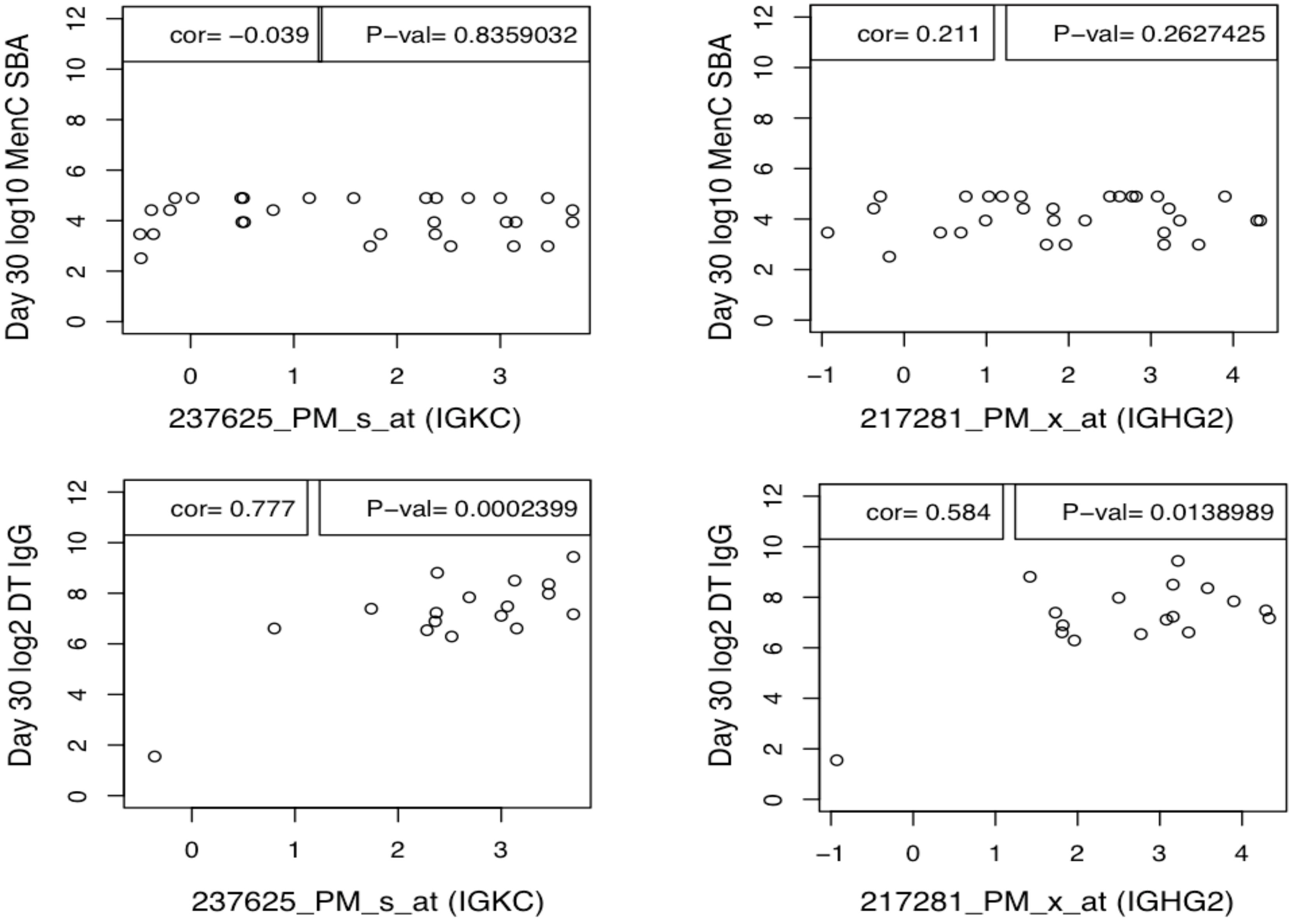


**Supplementary figure 9:** Correlation between the $log_2$ fold-change from day0 to day 7 in two differentially expression mRNAs (FDR < 0.05) and MenC-specific SBA titres 30 days after MenACWY vaccine. The diphtheria toxoid (DT) IgG plots only show individuals vaccinated with MenACWY-CRM. Pearson product-moment correlation coefficient (cor) and accompanying p-value are displayed.

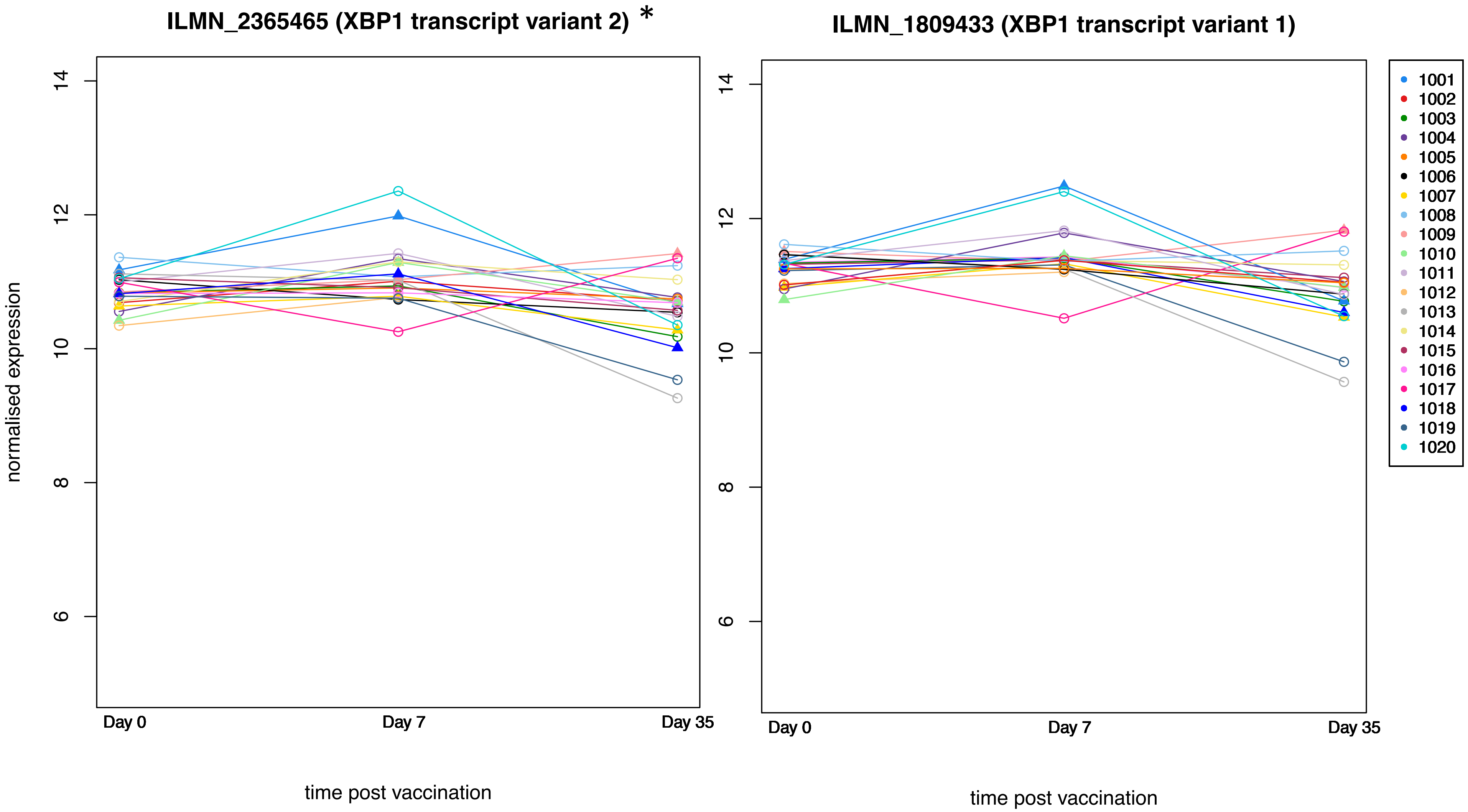


**Supplementary figure 10:** XBP1 transcripts levels measured by microarray. * annotated on microarray manifest as transcript variant 2 (i.e. spliced); however, probe sequence does not span spliced region, and is therefore, not specific to the spliced isoform. Triangles denote individuals whose first dose of MenACWY was the conjugate vaccine, and circles denote those whose first dose was the plain-polysaccharide MenACWY vaccine.

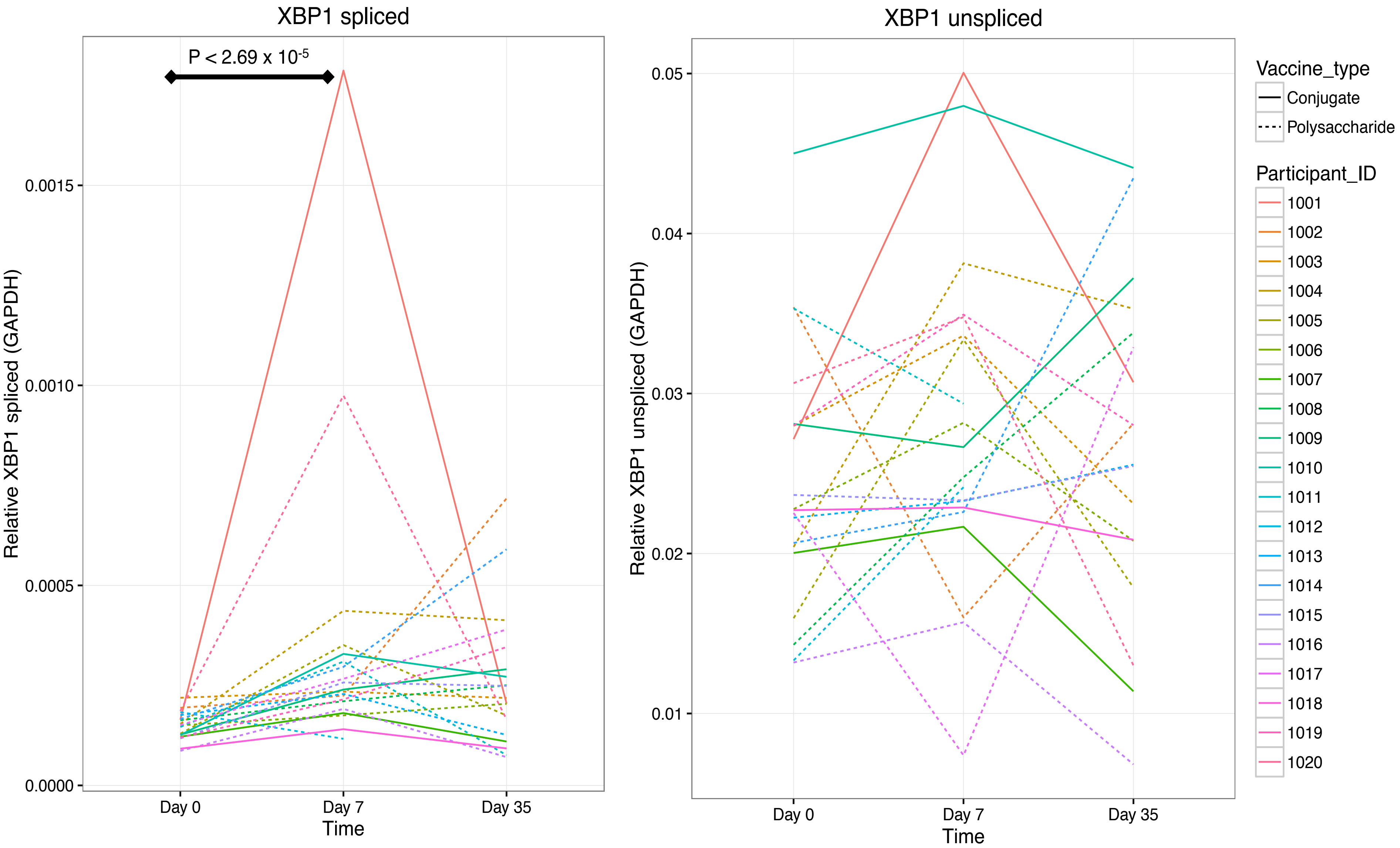


**Supplementary figure 11:** XBP1 transcripts levels measured by isoform-specific quantitative PCR.

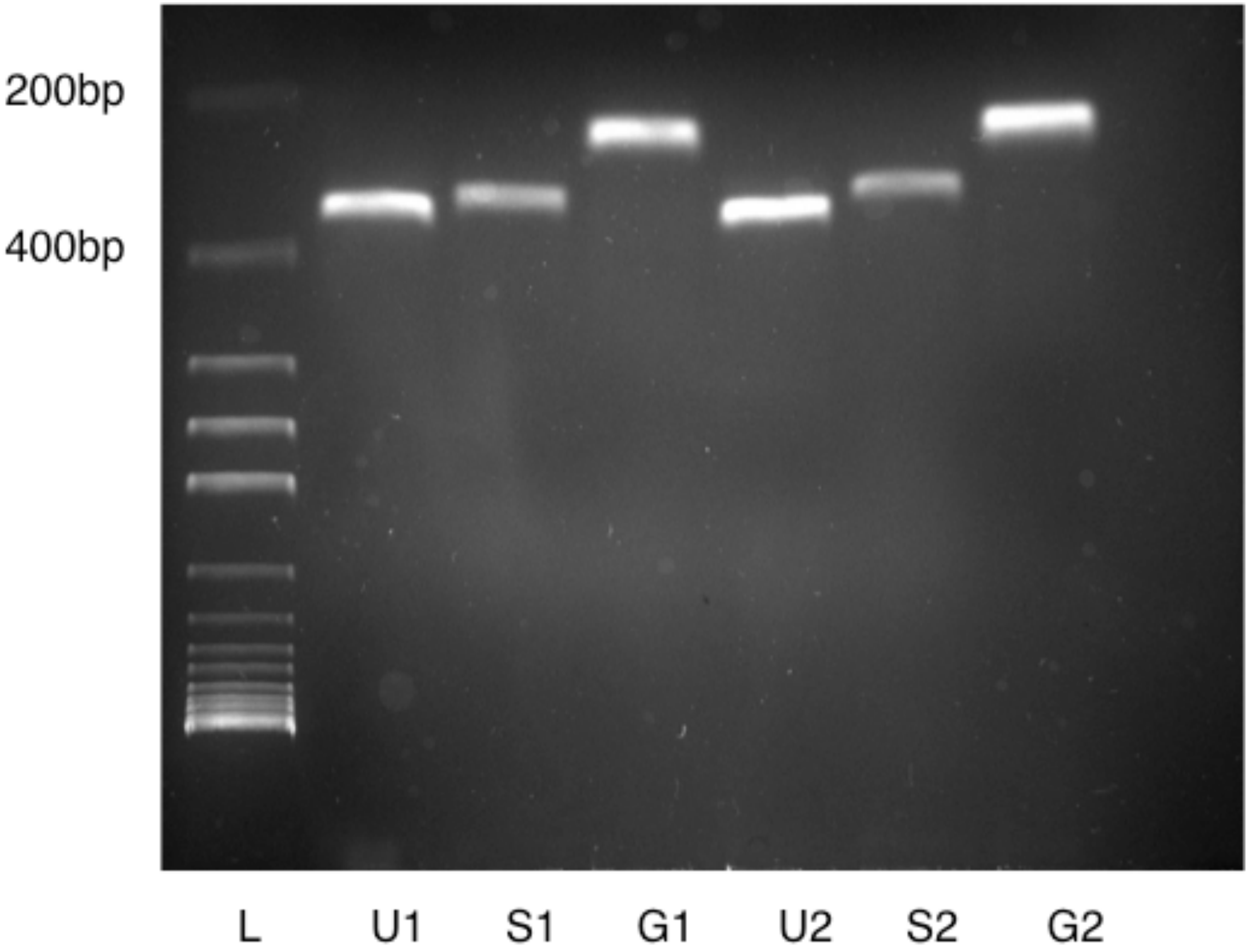


**Supplementary figure 12:** Agarose gel of *XBP1* RT-PCR amplicons. L= ladder; U1 and U2 are two different samples assayed using the “unspliced” *XBP1* primer set; S1 and S2 are two samples assayed using the “spliced” *XBP1* primer set; G1 and G2 are two samples assayed using the *GAPDH* internal control set.
